# Effects of markedness in gender processing in Italian as a heritage language: A speed accuracy tradeoff

**DOI:** 10.3389/fpsyg.2022.965885

**Published:** 2022-10-12

**Authors:** Grazia Di Pisa, Maki Kubota, Jason Rothman, Theodoros Marinis

**Affiliations:** ^1^Department of Linguistics, University of Konstanz, Konstanz, Germany; ^2^Department of Language and Culture, UiT The Arctic University of Norway, Tromsø, Norway; ^3^Department of Language and Education, Universidad Nebrija, Madrid, Spain; ^4^School of Psychology and Clinical Language Sciences, University of Reading, Reading, United Kingdom

**Keywords:** grammatical gender, heritage languages, Italian, markedness, speed-accuracy tradeoff

## Abstract

This study examined potential sources of grammatical gender variability in heritage speakers (HSs) of Italian with a focus on morphological markedness. Fifty-four adult Italian HSs living in Germany and 40 homeland Italian speakers completed an online Self-Paced Reading Task and an offline Grammaticality Judgment Task. Both tasks involved sentences with grammatical and ungrammatical noun-adjective agreement, manipulating markedness. In grammatical sentences, both groups showed a markedness effect: shorter reading times (RTs) and higher accuracy for sentences containing masculine nouns as compared to sentences with feminine nouns. In ungrammatical sentences, although both groups were sensitive to ungrammaticality, only HSs showed a markedness effect, that is, they had significantly longer RTs and higher accuracy when violations were realized on feminine adjectives. Proficiency in the HL was a significant predictor of accuracy and RTs at the individual level. Taken together, results indicate that HSs acquire and process gender in a qualitatively similar way to homeland native speakers. However, RT evidence seems to suggest that at least under particular experimental methods, markedness considerations are more prevalent for HSs resulting in a speed-accuracy tradeoff.

## Introduction

*Heritage speakers* (hereafter HSs) are early bilinguals who grow up using a language at home that is distinct from the majority language spoken in the society in which they are raised (see, e.g., Montrul, 2008; Rothman, 2009). In childhood, there is typically a significant shift in exposure from the heritage language (HL), usually coinciding with the start of school, to the societal majority language (ML). As a result, HSs often become dominant in the ML and their adult competence in the HL can vary considerably from homeland native speakers.

*Grammatical gender* (hereafter gender) is an inherent property of the noun reflected in agreement with other elements of the sentence (i.e., articles, determiners, and adjectives; Corbett, 1991). In most languages that have gender, assignment, and agreement are acquired early by monolingual children (*cf.* Chini, 1995 for Italian children; Müller, 1994 for French and German). Evidence from empirical research has shown that gender can be (particularly) vulnerable in heritage language acquisition (i.e., Montrul et al., 2008; Polinsky, 2008). However, there is also ample evidence showing that *some* HSs converge on a grammar for which gender is seemingly represented (and/or processed) in the same way it is for homeland speakers (see Alarcón, 2011; Bianchi, 2013; Kupisch et al., 2013; Van Osch et al., 2014; Irizarri van Suchtelen, 2016; Fuchs, 2019, 2021). Thus, HSs are fully capable of acquiring underlying syntactic gender systems; however, the ultimate representation of gender systems might not develop to be entirely the same as in homeland native speakers’ grammars. For example, HSs of Romance languages—where feminine is marked relative to the default masculine—tend to make more errors with feminine nouns (i.e., Montrul et al., 2008; Alarcón, 2011; Bianchi, 2013; Hur et al., 2020), suggesting that factors such as morphological markedness play a role.

It is also worth highlighting that much of what we know so far about the acquisition and processing of gender in adult HSs is based on behavioral offline methods (but see, e.g., Fuchs, 2019, 2021; Keating, 2022), such as acceptability judgement, comprehension, and recognition tasks. These provide significant insights into HS behavior. However, as recently pointed out by Bayram et al. (2021), offline methods alone can be problematic with regard to the kind of knowledge they are targeting, soliciting, and capturing. Behavioral tasks can be influenced by (unconscious and conscious) metalinguistic and affective variables. Since HSs are more likely to have less (and/or qualitatively distinct) metalinguistic knowledge (Rothman, 2007; Montrul et al., 2014; Bayram et al., 2019) and/or be more apprehensive to give definitive judgments (Polinsky, 2018), offline tasks alone could introduce noise that obscures HSs underlying competence.

With the above in mind, the main goal of the present study is to combine offline judgments with automatic processing responses (reaction times while reading) to determine whether HSs of Italian in the German context, like Romance homeland speakers (Alemán Bañón and Rothman, 2016; Alemán Bañón et al., 2017), are sensitive to morphological markedness when processing gender agreement violations during sentence comprehension.

### Gender in Italian and German

In Italian, there are two gender values: masculine and feminine. Gender assignment is largely transparent and follows both semantic and morpho-phonological rules. Canonical endings in Italian are-o and-a; thus, nouns ending in-o are typically masculine (*albero* “tree_M_”), while those ending in-a are usually feminine (*casa* “house_F_”; Schwarze, 2009). There are, of course, exceptions, for example, *problema*_M_ (“problem” ending in-a but masculine) and *mano*_F_ (“hand” ending in-o but feminine). Nouns with non-canonical endings are gender ambiguous and less frequent. In nouns ending in-e, for example, gender is not clearly marked, as these nouns could be either masculine (*pane*_M_ “bread”) or feminine (*notte*_F_ “night”). Nevertheless, some derivational suffixes can help to determine the gender of the noun, since they regularly co-occur with one of the two genders. For example, words that end in-trice and-zione (*calcolatrice*_F_ “calculator,” *posizione*_F_ “position”) are reliably feminine, whereas those ending in-a*le* and-one (*pugnale*_M_ “dagger,” *cotone*_M_ “cotton”) are masculine (Chini, 1995).

Italian requires gender agreement between the noun and its determiners, most modifying adjectives, and pronouns. In this study, we focus on the mastery of gender agreement on predicative adjectives; therefore, examples of gender agreement on adjectives are provided in (1) (a,b) for feminine nouns, and (c,d) for masculine nouns.

(1) a. *La*_F_ *luna*_F_ *rossa*_F_.

‘The red moon.’

b. *La*_F_ *volpe rossa*_F_.

‘The red fox.’

c. *Il*_M_ *libro*_M_ *rosso*_M_.

‘The red book.’

d. *Il*_M_ pesce *rosso*_M_.

‘The red fish.’

As shown in 1(b) and (d), the nouns *volpe* “fox” and *pesce* “fish” have no overt ending corresponding to feminine and masculine gender, as in 1(a) and (c). Rather, their lexical entries include a specification for feminine 1(b) and masculine 1(d) gender, respectively. In all cases, 1 (a–d) there is gender agreement between the definite article, the lexical gender feature of the head noun, and the agreeing (predicative) adjective.

As alluded to above, although reliable morphological marking in Italian is helpful, as in 1(a) and (c) above, all nouns have grammatical gender even in the absence of an unambiguous morphological ending on the noun, as in 1(b) and (d). Since gender is an inherent part of the noun’s entry in the mental lexicon, it brings together lexical and syntactic aspects (Corbett, 1991; Kramer, 2015). At the lexical level, learners need to first assign gender to nouns (*assignment*); then, when used (or processed) in a sentential context, the syntactic reflexes of agreement come to bear (*agreement on adjectives*).

Unlike Italian, German has a three-way gender system with masculine, feminine, and neuter nouns (Durrell, 2011). With respect to gender assignment, nouns are largely opaque. Even though there are some semantic, morphological, and phonological patterns, there are also many exceptions (Köpcke, 1982). This makes the German system much less transparent compared to the Italian one.

As for agreement, in contrast to Italian, gender in German is not marked on the noun itself, but rather on determiners and adjectives occurring within the same DP (or referring to it elsewhere). However, the gender of determiners and adjectives can sometimes be ambiguous since agreement also depends on definiteness (definite vs. indefinite), case (nominative, accusative, dative, and genitive), and number (singular vs. plural; Kunkel-Razum et al., 2009).

### Grammatical gender in heritage speakers

In the last two decades, HSs knowledge of gender systems has been the object of considerable research. Within the available literature there is a juxtaposition of findings, sometimes even for the same HL (e.g., Spanish) depending on the study/method used. While some show that HSs struggle with gender assignment and/or subsequent agreement in production and comprehension in various HLs (i.e., Russian: Polinsky, 2008; Spanish: Montrul et al., 2008), others demonstrate that HSs do not differ qualitatively from homeland native speakers (Italian: Bianchi, 2013; French: Kupisch et al., 2013; Spanish: Alarcón, 2011; Montrul et al., 2013; and Russian: Laleko, 2018). This suggests that mastery of gender systems in HLs that are qualitatively the same as in homeland varieties in HLs is attainable, although they can be vulnerable under specific conditions.

A closer look at these studies shows that gender in HSs is significantly affected by the HL-ML combination, the level of HL proficiency, the HL use, and the age of onset (AoO) of bilingualism. Most of the studies investigating gender have tested HSs whose ML was a non-gendered language, most often English (i.e., Spanish: Montrul et al., 2008; Russian: Polinsky, 2008). Fewer studies have been conducted in language pairs in which both languages have gender, but differ with respect to the properties of their gender systems (i.e., Italian and German: Bianchi, 2013; French and German: Kupisch et al., 2013). Regarding gender and proficiency, findings are controversial with some studies reporting higher error rates for HSs with lower proficiency level (i.e., Montrul et al., 2008), and other studies testing HSs with a higher level of proficiency finding no differences in terms of performance between HSs and matched homeland native speakers (i.e., Alarcón, 2011; Bianchi, 2013; Kupisch et al., 2013). However, even advanced HSs often are different compared to monolinguals with respect to grammatical gender when tested on non-canonical nouns (i.e., Bianchi, 2013; Montrul et al., 2013). Previous studies measuring HSs’ relative amount (and quality) of exposure and use of their HL have shown that variation in HL exposure has consequences for HL development in children (i.e., Gagarina and Klassert, 2018; Torregrossa et al., 2021) and maintenance in adults (i.e., Lloyd-Smith et al., 2019, 2020). Some studies on gender have shown that HL exposure and/or use has an effect on HSs’ performance (i.e., Bianchi, 2013); however, others (i.e., Fuchs, 2021) found no evidence. Therefore, it is still an open question to what extent one’s individual experiences with the HL modulate gender processing in HSs. Furthermore, previous studies have shown that AoO of bilingualism plays a role in the acquisition and maintenance of HLs, usually leading to more variable outcomes in simultaneous bilinguals (i.e., Montrul, 2008; Montrul et al., 2014; Giancaspro, 2017). However, few studies on gender in adult HSs have examined effects of AoO of bilingualism revealing controversial results (for Italian: Bianchi, 2013; for Spanish: Montrul, 2008; Keating, 2022), thus leaving open the question of the extent to which the syntax of gender is really affected by AoO.

In line with the inconsistent findings of the above factors, and in light of recent turns in various literature examining bilingual language and cognitive systems that advocate for regressing factors pertaining to exposure and, crucially, dynamic engagement with language in various contexts (DeLuca et al., 2019; Titone and Tiv, 2022), we collected detailed information on all these factors. The logic in doing so is to be able to unpack the conditions under which general observations are more or less true. In other words, it could be the case, for example, that morphological markedness affects HS processing more or less under specific conditions for individual HSs.

### Morphological markedness

As it is the case that markedness can be understood differentially (morphologically, semantically, and frequency based), let us start by being explicit as to what we take to be marked and why in the present context. We take the position that in Italian gender, feminine is marked relative to masculine. Given the robust associations that the classical morphemes (-*o*, -*a*, -*i*, and -*e*) have with their respective gender, in one sense of markedness, it would be reasonable to argue that each is equally morphologically marked. This, however, is not the sense we mean. Claiming that feminine is marked and, relatedly, that masculine is the default is supported by both a frequentist position and various facts. In Italian, masculine nouns by far outnumber feminine ones: 60% are masculine and 40% are feminine (Costa et al., 2003). Furthermore, when one considers some classical diagnostics, it is easy to see how masculine is the default. For example, when nominalizing (and/or conceptually abstracting) anything new or novel in Italian, masculine is the gender assigned (D'Achille, 2003) as shown when a verb is made into a noun: *Il*_M_/*La_F_ *fumare è dannoso alla salute* “Smoking is harmful to health.” Another example is the case of lexical borrowings that mostly take the masculine gender whether or not they are incorporated into Italian morpho-phonological or remain as lexical insertions, for example, *il film, il software, lo smartphone.*

Morphological markedness theory (Battistella, 1990) assumes that feature values, e.g., masculine vs. feminine for gender, are asymmetrically represented and have a hierarchical structure, with the more general or *unmarked* element (masculine in the Romance case) being the “default value,” indicating just the presence of a grammatical feature (gender), and the most specific or *marked* version(s) (feminine in Romance languages) indicating a specific feature value (or specification; Battistella, 1990). In Italian, masculine is the most frequent gender (Cacciari, 2011) and it is also the least-marked; thus, masculine is considered the “default gender,” while feminine is regarded as marked (D'Achille, 2003). In German, however, the default gender is not as clear considering the presence of a third gender (neuter) in its system; however, convincing evidence exists to suggest that masculine is also the default gender in German (e.g., Steinmetz, 2006).

Previous research on homeland native speakers and L2 speakers examining noun–adjective agreement in Romance languages like French (Vigliocco and Franck, 1999, 2001), Italian (Vigliocco and Franck, 2001), and Spanish (e.g., Antón-Méndez et al., 2002; McCarthy, 2008) has shown that agreement errors were more frequent when the head noun was feminine (marked). This tendency to overuse the default gender (masculine) on agreement targets suggests the use of masculine as a default agreement strategy (i.e., McCarthy, 2008). Furthermore, some studies have shown that agreement violations realized on marked elements are detected more easily, consistent with the claim that marked features are more disruptive, thus more costly to process and consequently more recognizable during the processing of agreement (e.g., Deutsch and Bentin, 2001; Nevins et al., 2007).

In a recent set of neuroimaging studies, Alemán Bañón and Rothman (2016) and Alemán Bañón et al. (2017) found that both homeland Spanish native speakers and Spanish L2 learners were sensitive to markedness asymmetries, such that the P600 for gender violations emerged earlier and it was larger for *feature clash (marked) errors* (masculine noun+*feminine adjective) than *default (unmarked) errors* (feminine noun+*masculine adjective). This is consistent with the possibility that errors that involve mismatching marked features are more disruptive and easily detectable. While the two groups differed quantitatively, neither showed any systematic evidence of reliance on morphological defaults, although their online processing was sensitive to markedness in a native-like manner.

Previous studies focusing on the linguistic factors underlying gender errors in HSs have reported the tendency for HSs to be more accurate on gender assignment and agreement with the language-specific unmarked form, for example, in Spanish masculine nouns compared to feminine ones (i.e., Montrul et al., 2008; Van Osch et al., 2014; Irizarri van Suchtelen, 2016; Goebel-Mahrle and Shin, 2020; Hur et al., 2020). This over-reliance on the masculine in the above cases could be explained in terms of morphological markedness. Nevertheless, as the tasks used in previous studies were offline, we do not know how (or if) markedness affects online sentence processing.

Depending on the research question, homeland native comparisons are not always necessary or particularly illuminating in HS studies. Herein, however, we are interested in the comparison for a few reasons. To begin, we do not know if markedness matters for online gender agreement processing with this type of method in any group—the studies we referenced showing such effects in homeland native language processing are not reading RT studies. While we realize that homeland Italian speakers are not necessarily the baseline for our Italian HSs, it would be interesting to see the extent to which markedness plays a role for the homeland group with this method to best contextualize/interpret what we observe for the present HSs. There is good reason to anticipate that HSs will show considerable markedness effects, above and beyond what the homeland speakers may or may not show, precisely because HS grammars have been shown to be particularly reliant, if not magnify (morphological) defaults (Polinsky, 2018). If so, in the present context, one might expect marked agreement (a)symmetries to be even more salient for HSs.

### Research questions and hypotheses

Given the previous discussion, the present study aims to answer the following research questions:

*RQ1: Are HSs sensitive to morphological markedness, and if yes, how does markedness affect the processing of agreement violations in HSs as compared to homeland speakers?*

Based on previous research (Alemán Bañón and Rothman, 2016), we expect homeland speakers to be sensitive to morphological markedness (feature clash being more marked: masculine noun+*fem. adjective). Behaviorally, evidence in support of this would be obtained if they are more accurate with feature clash errors than default ones, although given that accuracy is assessed *via* offline judgment there could be a ceiling effect in accuracy. Conversely, in terms of the online measure, we would definitely expect sensitivity to markedness shown *via* speakers’ slowing down with feature clash errors, indicating their grammatical system has detected an error. Regarding specific error type, we would expect RT slowdowns in the SPRT and higher accuracy in the Grammaticality Judgment Task (GJT) for feature clash (marked) errors (masculine noun+*feminine adjective), as opposed to no RT slowdowns and lower accuracy for default (unmarked) errors (feminine noun+*masculine adjective). As this is the first study to test markedness in this domain in HSs, we are unsure what to expect precisely although there is no reason, *a priori*, to not expect them to be equally sensitive to markedness. After all, we know that other sets of bilinguals are, even non-natives ones (Alemán Bañón et al., 2017). We might expect Italian HSs to be over-reliant on defaultness (masculine as default gender) as well as more sensitive to feature clash (marked) errors as compared to default (unmarked) ones given the heightened role that defaults can play in HS grammatical systems (Polinsky, 2018).

*RQ2: Do proficiency and extra-linguistic factors (i.e., type of bilingualism, quantity and quality of input) affect accuracy and RTs in HSs?*

In order to understand whether specific background variables affect HSs’ performance in both tasks, we will consider the variables that have been shown to affect HL acquisition (HL proficiency, HL use in the home and in the society, type of bilingualism—simultaneous vs. sequential). We expect HSs’ overall performance to benefit from higher proficiency in the HL (i.e., Bianchi, 2013; Kupisch et al., 2013) and more HL use (i.e., Bianchi, 2013). Regarding AoO, there are two possible scenarios: in line with Montrul (2008) and Keating (2022), sequential HSs could be more accurate and show sensitivity to markedness earlier (faster RTs) than simultaneous HSs; or similar to Bianchi (2013), we could find no difference between the two groups of HSs due to the fact that gender acquisition in Italian is not problematic, given the high degree of transparency of the Italian gender system (Kupisch et al., 2002; Velnić, 2020), and thus robust to AoO of bilingualism effects.

*RQ3: Is HSs’ use of markedness information during processing of agreement affected by task modality (offline vs. online)?*

We expect to find an effect of markedness in both tasks; however, we leave open the possibility that the degree of this effect will differ across the two modalities.

To answer these questions, we tested a group of HSs of Italian living in Germany and a group of homeland Italian native speakers living in Italy. We used a Self-Paced Reading Task (SPRT) to tap into implicit processing of ungrammaticality and judgments from a Grammaticality Judgment Task (GJT) to examine accuracy, the latter potentially tapping into more explicit factors. The tasks presented complex sentences in Italian where markedness was examined by systematically manipulating the gender specification of the agreeing adjective following the noun.

## Materials and methods

### Participants

Fifty-four adult HSs of Italian (35 females, *M_age_* = 28.15; *SD* = 6.20; *range* = 18–41) living in Germany and 40 adult homeland Italian speakers (29 females, *M_age_* = 25.65; *SD* = 3.99; *range* = 18–39) living in Italy participated in the study. We initially recruited 55 HSs but one participant was excluded because exposed to three languages from birth. All the homeland speakers grew up monolingually in Italy and were living in Italy at the time of testing. All HSs grew up in Germany; however, six HSs were not born in Germany (five were born in Italy and one was born in Argentina), but even in these cases, each had moved to Germany between the age of 1 and 5 years (*M_age_* = 2.5; *SD* = 1.64). The heritage group comprised 33 simultaneous bilinguals who were exposed to German from birth and had one Italian and one German-speaking parent and 21 sequential bilinguals who had two Italian-speaking parents and their first intensive contact with German occurred between 3 and 6 years (*M_age_* = 1.5; *SD* = 1.97) when they started kindergarten in Germany. They all completed their schooling in Germany and they were living in Germany at the time of testing. To assess the effect of HL use on the processing of gender in Italian as well as to quantify aspects of Italian use across the lifespan, all participants completed the Language and Social Background Questionnaire (LSBQ; Anderson et al., 2018). The LSBQ aims at capturing participants’ second language use from childhood to the present day and across several settings and dimensions. It yields two scores related to the amount of (bilingual) language use within specific communicative settings. Specifically, the social score (in our study referred as “HL in the society”) is related to language use in the participant’s social life (e.g., at work, when writing emails, watching TV, etc.), the possible range is: –7.5 to 80.304; the higher the score, the more frequently the second language is used in social settings. Whereas the home score (in our study referred as “HL in the home”) is related to language use in home settings (for instance language used with grandparents, during infancy, proficiency in the second language, etc.); the possible range is: –13.9 to 24.163, the higher the score, the more the second language is used in home settings. Detailed demographic information about the participants is provided in Supplementary Table S1 in the Supplementary material.

### Proficiency

**Figure 1:**
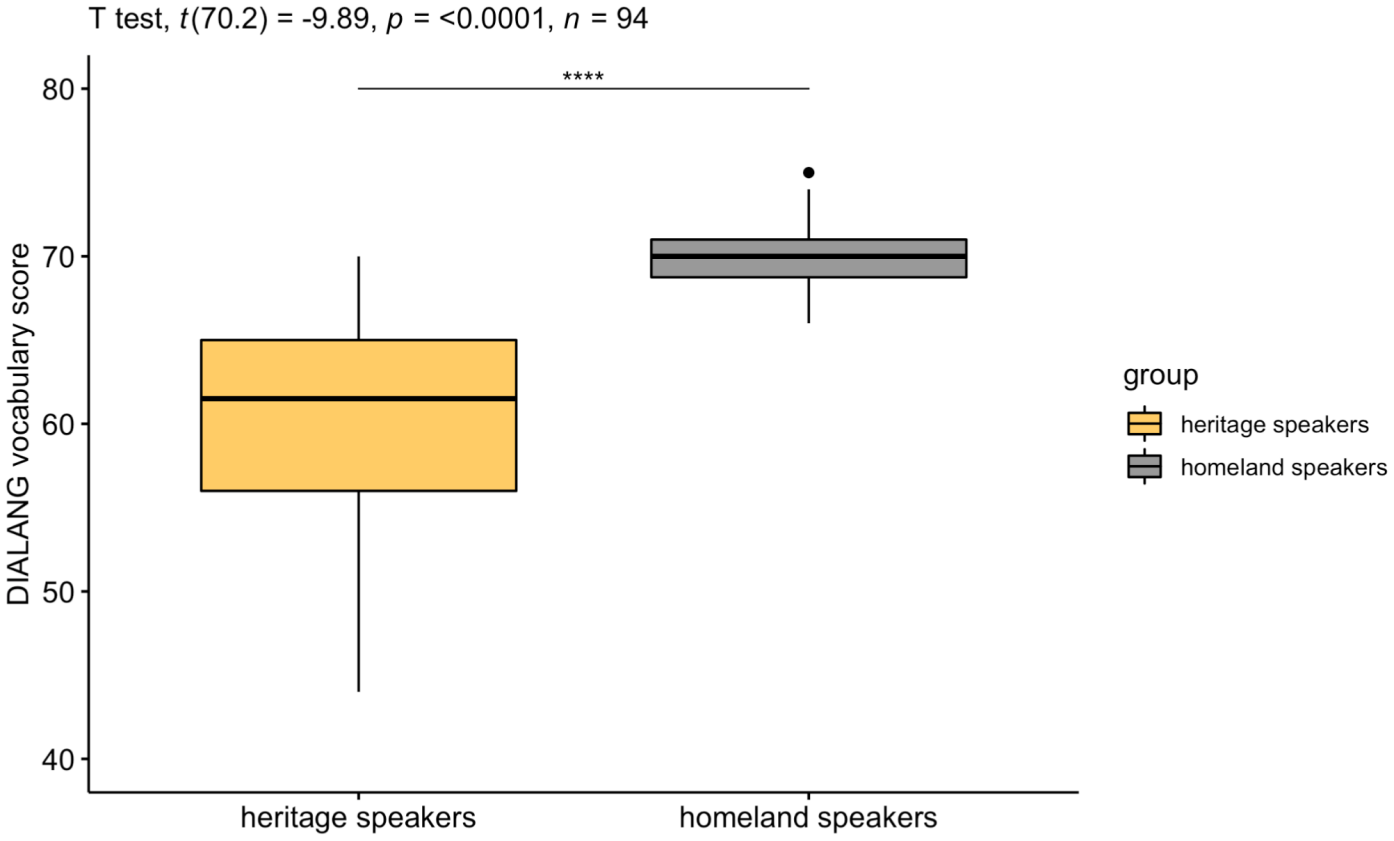
Heritage and homeland speakers’ scores on the Italian vocabulary test DIALANG (raw scores).

Proficiency in Italian was assessed using an adapted version of the Italian placement test originally created by Alderson (2005), known as DIALANG test battery. The test consists of 50 real words and 25 pseudo-words requiring a YES or NO response. In our adaptation (see Lloyd-Smith et al., 2021), the items appeared in the center of the screen one at the time, and participants were instructed to press on their keyboard key F if they thought the word existed or key J if they did not. Scoring consisted of simply the sum of all correct answers (i.e., one point for each correctly identified word or non-word). The maximum possible score was 75. As shown in Figure 1, HSs had lower proficiency and their scores displayed a much larger degree of variation (*M* = 60.33; *SD* = 6.49; *range* = 44–70) as compared to the homeland native speakers (*M* = 69.80; *SD* = 2.33; *range* = 66–75).

### Materials

The study included two main experimental tasks: a *self-paced reading task* and a *grammaticality judgment task*. The materials for both tasks comprised 80 sentences of eight words each. All sentences presented the same structure: subject + auxiliary verb *have* + past participle + indefinite article + trigger noun + adjective (always in post-nominal position) + preposition + object. Morphological markedness was manipulated in the gender specification of the trigger noun and the agreeing adjective, as shown in Table 1, which provides a sample of each of the four experimental conditions. The trigger noun was feminine in half of the sentences (*N* = 40) and masculine in the other half (*N* = 40). This can be seen by comparing the sentences in (1–2) to those in (3–4) in Table 1. In (1–2), the noun *Torre* “tower” is feminine and therefore, the agreeing adjective *antica_FEM_* “old” must also be feminine, as shown in (1). Otherwise, the string would be ungrammatical *antico_MASC_*, as shown in (2). The opposite pattern is shown in (3–4), where the trigger noun *pesce* “fish” is masculine and, therefore, the agreeing adjective *rosso_MASC_* “red” must also be masculine, as shown in (3). Otherwise, the string would be ungrammatical, as shown in (4).

**Table 1:** Sample stimuli for the experimental conditions.

FEMININE NOUN
**Grammatical feminine**
1. Daniele ha fotografato una torre antic**a** a Roma.
Daniele took a picture of a tower_-FEM_ old_-FEM-marked_ in Rome.
**Ungrammatical feminine - Default (Unmarked) Error**
2. Daniele ha fotografato una torre *antic**o** a Roma.
Daniele took a picture of a tower_-FEM_ old_-MASC-unmarked_ in Rome.
**MASCULINE NOUN**
**Grammatical masculine**
3. Alessandro ha comprato un pesce ross**o** alla fiera.
Alessandro bought a fish_-MASC_ red_-MASC-unmarked_ at the fair.
**Ungrammatical masculine - Feature Clash (Marked) Error**
4. Alessandro ha comprato un pesce *ross**a** alla fiera.
Alessandro bought a fish_-MASC_ red_-FEM-marked_ at the fair.

The study also included 80 filler sentences (40 grammatical, 40 ungrammatical) which did not manipulate gender agreement and did not include any adjectives. The overall design encompassed an equal amount of grammatical and ungrammatical sentences. These 160 sentences were counterbalanced across four experimental lists where the carrier sentences were the same. Each participant was pseudorandomly assigned to one of the four lists (the same list was used in the SPRT and in the GJT), so that a given participant would see 20 items per each of the two conditions in (1–2; a total of 40) and 20 items per each of the two conditions in (3–4; a total in 40), but no participant saw the same sentence twice.

### Properties of the stimuli

None of the trigger nouns exhibited the-o/*-a* canonical endings strongly associated with masculine and feminine genders in Italian to ensure that participants could not resort to a phonological strategy for matching the agreeing elements. Instead, we selected nouns ending in-e that in Italian are either completely opaque with respect to their gender or the-e forms part of a derivational suffix which defaults to one or the other gender. In sum, we controlled for noun ending transparency, so that half of the trigger nouns were truly opaque; hence, gender could not be recovered from the surface form (e.g., *pont-e*_M_ “bridge”), while the other half consisted of nouns ending with *-e* as part of a derivational suffix providing a cue about gender, making them more transparent (e.g., *magli-one*_M_ “jumper”). Gender congruency was also controlled in a way that half of the trigger nouns in Italian share the same gender in German, while the other half have the opposite gender. We only used nouns with masculine or feminine gender; thus, we did not use nouns that were neuter in German. Furthermore, the trigger nouns were presented in both the singular and the plural form always counterbalanced. A total of 77 trigger nouns were used: 39 masculine nouns (one noun was used twice) and 38 feminine nouns (two nouns were used twice), see Supplementary material 1 for a complete list of all the trigger nouns. A log frequency count for all nouns and adjectives was obtained from the CoLFIS corpus (Corpus e Lessico di Frequenza dell’Italiano Scritto, Bertinetto et al., 2005). Masculine and feminine nouns were matched with respect to frequency, *t*(75) = - 0.446, *p* > 0.1, and number of syllables, *t*(75) = -0.609, *p* > 0.1. The masculine and feminine versions of the adjectives were also matched for frequency, *t*(78) = 0.803, *p* > 0.1, and number of syllables, *t*(78) = 1.028, *p* > 0.1.

### Tasks

#### Self paced reading task

The SPRT used a non-cumulative word-by-word center presented design (Marinis, 2010). The 160 sentences were divided into four blocks of 40 sentences each, with 20 correct and 20 incorrect sentences per block separated by short breaks. Detailed instructions and four practice sentences with accuracy feedback preceded the experiment to familiarize participants with the task. None of the practice trials involved agreement errors. In addition, in order to avoid repetition effects, the practice sentences were designed with lexical material that did not appear in the experimental stimuli. Immediately after the practice, the main experiment began. The sentences were presented randomly.

Each trial began with a fixation cross and the first word appeared after 500 ms. Participants used the spacebar to advance through the words. To ensure that the participants were paying attention to the stimuli, a binary yes/no comprehension question appeared after 35% of the sentences on a separate display screen [see (2) for a sentence example]. Participants responded using keys F (YES) or J (NO) on their keyboard. The question stayed on the screen until the participant answered. No feedback was given for correct or incorrect answers. Participants were instructed to read the sentences as fast as possible, and they were told that the task targeted reading comprehension.

(2) Daniele | ha | fotografato | una_F_ | torre_F_ | antica_F_ | a | Roma.

R1 | R2 | R3 | R4 | R5 | R6 | R7 | R8.

*Daniele è stato a Roma?*

‘Was Daniele in Rome?’

a. *Si* “Yes.”

b. *No* “No.”

#### Grammaticality judgement task

The GJT stimuli were identical to those of the SPRT, with 160 sentences divided into four blocks of 40 sentences each, with 20 correct and 20 incorrect sentences per block separated by short breaks. Participants read the full sentence on the screen and were asked to judge whether or not the sentence was grammatically correct by pressing keys F (YES) or J (NO) on their keyboard. All sentences were presented in a random order.

#### Gender assignment task

In order to check whether participants assigned the correct gender to the target nouns used in the main tasks, participants completed a Gender Assignment Task (GAT). Participants were presented with all 77 trigger nouns from the experimental sentences and were instructed to select the appropriate gender-marked determiner from among two options (*il*_M_ “the” vs. *la*_F_ “the”) by using the keys F (*il*) and J (*la*) on their keyboard. The trigger nouns were presented one after the other in isolation, and at the end of the task, participants were also asked to indicate whether they knew each word and its meaning.

### Procedure

Due to the pandemic, the experimental session was completed online *via* the internet by each participant using their personal computer. All tasks were created using Gorilla Experiment Builder (www.gorilla.sc; Anwyl-Irvine et al., 2020). Data were collected between 28 June 2020 and 30 September 2020. Prior to the experiment, participants filled out the language and social background questionnaires, then they completed the DIALANG in Italian, the SPRT, the GJT, and the GAT. The entire session lasted approximately 45 min and participants were allowed to have breaks in between the tasks. Participants received a compensation for their participation. All participants provided informed consent to take part in the study and all procedures were approved by the research ethics committee of the University of Konstanz, Germany.

### Analyses

Trials containing trigger nouns reported as “unknown” by the participants were removed from all the tasks. Homeland speakers reported knowing all the trigger nouns, while HSs’ knowledge of trigger nouns was high with some variability (*M* = 72 out of 77; *SD* = 4.65; *range* = 57–77). Furthermore, accuracy on the GAT was used for data cleaning in the SPRT and in the GJT for both groups; thus, we only included trials with nouns for which the participants assigned the correct target gender in the GAT. Moreover, raw RTs were screened for extreme values and outliers (Keating and Jegerski, 2015; Marsden et al., 2018). We excluded all segments with RTs below 150 ms and above 6,000 ms on the basis of histograms. For the remaining data, we trimmed all raw RTs that deviated more than 2.5 SDs below and above from the participants’ mean per position and per condition. Percentages of removed data and final data pool are provided in Supplementary Table S2 in the Supplementary material.

Sentences were segmented into eight regions (see Example 2 above) and the analyses for RTs were done on three specific regions of interest: *Region 5* = noun (pre-critical), *Region 6* = adjective (critical region), and *Region 7* = spill-over (post-critical).

Accuracy data from the GJT were analyzed with mixed effects logistic regressions (Jaeger, 2008), and RTs from the SPRT were analyzed with mixed effects linear models (Baayen et al., 2008) in R (R Core Team, 2016). We used the mixed function in the *afex* package (Singmann et al., 2022) to run a likelihood ratio test. The categorical variables were sum-coded and numerical variables were centered around the mean. Pairwise *post-hoc* comparisons with Tukey’s contrasts were conducted using the *emmeans* package (Lenth, 2022). Figures were produced using the package *ggplot2* (Wickham, 2016).

The first analysis focused on the comparison between HSs and homeland speakers to establish whether both groups were sensitive to markedness (RQ1) in terms of accuracy in the GJT and RTs in SPRT. The dependent variable was a binary outcome (correct or incorrect) for the GJT and RTs for the SPRT. We included *Group* (heritage vs. homeland speakers), *Grammaticality* (grammatical vs. ungrammatical), and *Gender* (feminine vs. masculine), as well as their interactions (*Group:Grammaticality, Group:Gender, Grammaticality:Gender,* and *Group:Grammaticality:Gender*) as fixed effects. We included Grammaticality*Gender slopes for Subject and Item intercepts and simplified the model following Bates et al. (2015) until there were no convergence issues.

The second analysis was restricted to the heritage group in order to investigate to what extent proficiency and extra-linguistic factors predicted the likelihood of accuracy as well as RTs (RQ2). *Grammaticality* (grammatical vs. ungrammatical), *Gender* (feminine vs. masculine), the DIALANG proficiency scores, and type of bilingualism (simultaneous vs. sequential), HL use in the home (HL home) and in the society (HL society) as well as their interactions (*Grammaticality:Gender, Grammaticality:Proficiency, Grammaticality:Bilingualism, Grammaticality:HL_home, Grammaticality:HL_society, Gender:Proficiency, Gender:Bilingualism, Gender:HL_home, Gender:HL_society, Grammaticality:Gender:Proficiency, Grammaticality:Gender:Bilingualism, Grammaticality:Gender:HL_home,* and *Grammaticality:Gender:HL_society*) were included as fixed effects in the model. The proficiency scores as well as HL home and HL Society scores were centered prior to statistical analyses. We included Grammaticality*Gender slopes for Subject and Item intercepts and simplified the model until there were no convergence issues.

## Results

Figures and averages of the SPRT are shown in raw measures for ease of interpretation, but the models were fit to log-transformed RTs, to remove skew, and to normalize model residuals (Vasishth and Nicenboim, 2016). Accuracy results from the GAT are provided in Supplementary material 4.

### Self paced reading task

**Figure 2:**
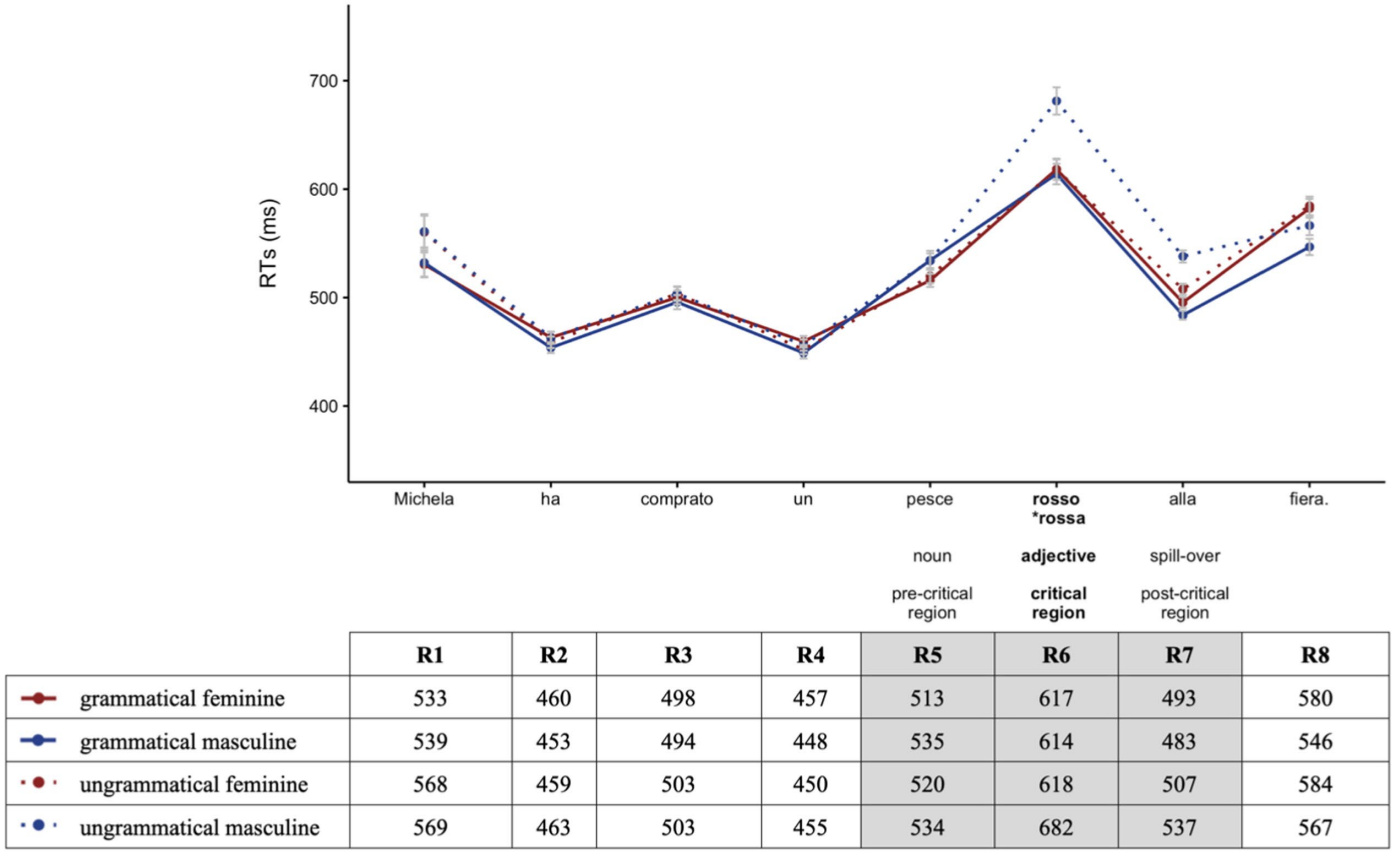
Heritage speakers (HSs’) mean reading times (RTs) by region for grammatical (solid lines) vs. ungrammatical (dotted lines) sentences for feminine (red) and masculine (blue).

**Figure 3:**
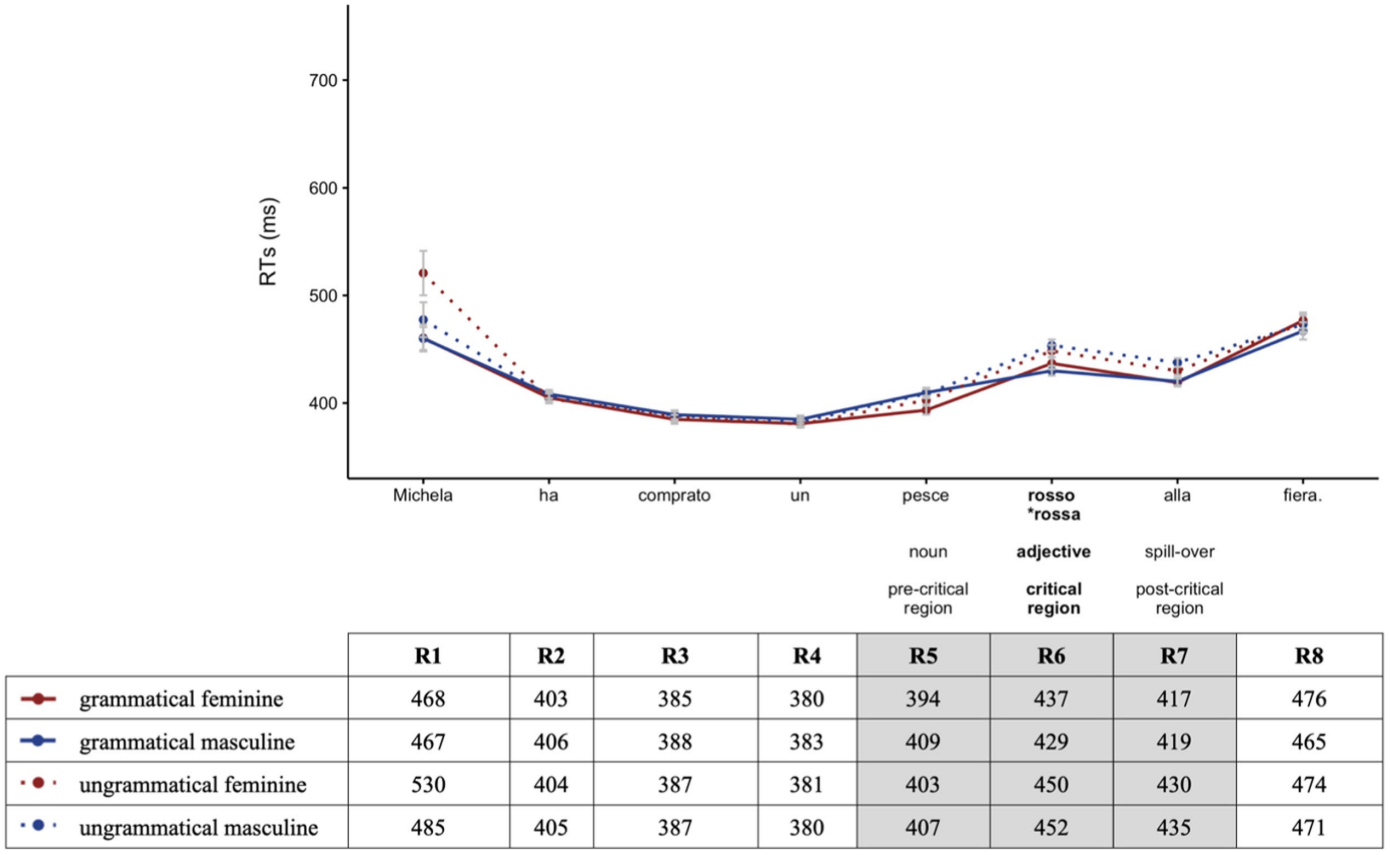
Homeland speakers’ mean RTs by region for grammatical (solid lines) vs. ungrammatical (dotted lines) sentences for feminine (red) and masculine (blue).

Before analyzing participants’ RT data, we examined accuracy rates for the comprehension question responses. Both groups demonstrated a high mean accuracy rate: 93.0% (*SD* = 0.25) in the heritage and 95.2% (*SD* = 0.21) in the homeland speaker group. Thus, participants were reading for meaning and were attentive during the task. All participants scored above 50% accuracy, so no participant was excluded. For the analysis of RTs, we only included trials that received correct answers. As shown in Figures 2, 3 illustrating overall reading patterns (non-log-transformed RTs), HSs had longer RTs than homeland speakers.

In Region 5 (pre-critical) containing the noun, the significant effect of *Group* (Chisq = 19.74, *p* < 0.001) indicates that overall HSs had longer RTs as compared to homeland speakers. We found no effect of *Grammaticality* nor *Gender*, indicating that the effects observed in the critical region did not start earlier. No further analyses were conducted on this region.

In Region 6 (critical), the between-group analysis revealed a significant effect of *Grammaticality* (Chisq = 5.76, *p* = 0.016), indicating that both groups had shorter RTs for grammatical sentences compared to ungrammatical ones. The effect of *Group* (Chisq = 27.67, *p* < 0.001) indicates significantly longer RTs for HSs as compared to homeland speakers. The significant interaction between *Group*:*Gender* (Chisq = 5.22, *p* = 0.022) and *post-hoc* pairwise comparisons indicates that the difference in RTs between feminine and masculine nouns was significantly different between groups (*β* = -0.040, *SE* = 0.017, *z* = -2.327, *p* = 0.019), indicating that HSs had shorter RTs for feminine nouns compared to masculine ones in comparison to homeland speakers. The three-way interaction between *Group:Grammaticality:Gender* was not significant (Chisq = 2.78, *p* = 0.096). However, since this was our *a priori* comparison, we ran *post-hoc* pairwise comparisons, showing that the difference in RTs between feminine and masculine nouns in the grammatical conditions was not different between HSs and homeland speakers (*β* = -0.014, *SE* = 0.023, *z* = -0.607, *p* = 0.544). In the ungrammatical condition, however, the difference in RTs between feminine and masculine nouns was significantly different between groups (*β* = -0.067, *SE* = 0.023, *z* = -2.843, *p* = 0.005), indicating that HSs had shorter RTs with feminine nouns (where the ungrammaticality was caused by an unmarked masculine adjective) compared to masculine nouns (where the ungrammaticality was caused by a marked feminine adjective).

In Region 7 (spill-over), a significant effect of *Grammaticality* (Chisq = 26.36, *p* < 0.001) indicates shorter RTs for the grammatical conditions compared to the ungrammatical ones in both groups. The significant main effect of *Group* (Chisq = 21.98, *p* < 0.001) reflects overall longer RTs for HSs as compared to homeland speakers. The significant interaction between *Group:Grammaticality:Gender* (Chisq = 5.94, *p* = 0.015) indicates that grammaticality affected RTs in HSs and homeland speakers in a different way. Subsequent *post-hoc* pairwise comparisons showed that in the grammatical conditions, the difference in RTs between feminine and masculine nouns was not different between HSs and homeland speakers (*β* = -0.014, *SE* = 0.023, *z* = -0.60, *p* = 0.543). However, the difference in RTs between feminine and masculine nouns in the ungrammatical conditions was significantly different between groups (*β* = -0.066, *SE* = 0.023, *z* = -2.843, *p* = 0.004), indicating that for HSs ungrammaticality with an unmarked adjective (feminine ungrammatical) led to shorter RTs than ungrammaticality with a marked adjective (masculine ungrammatical).

**Figure 4:**
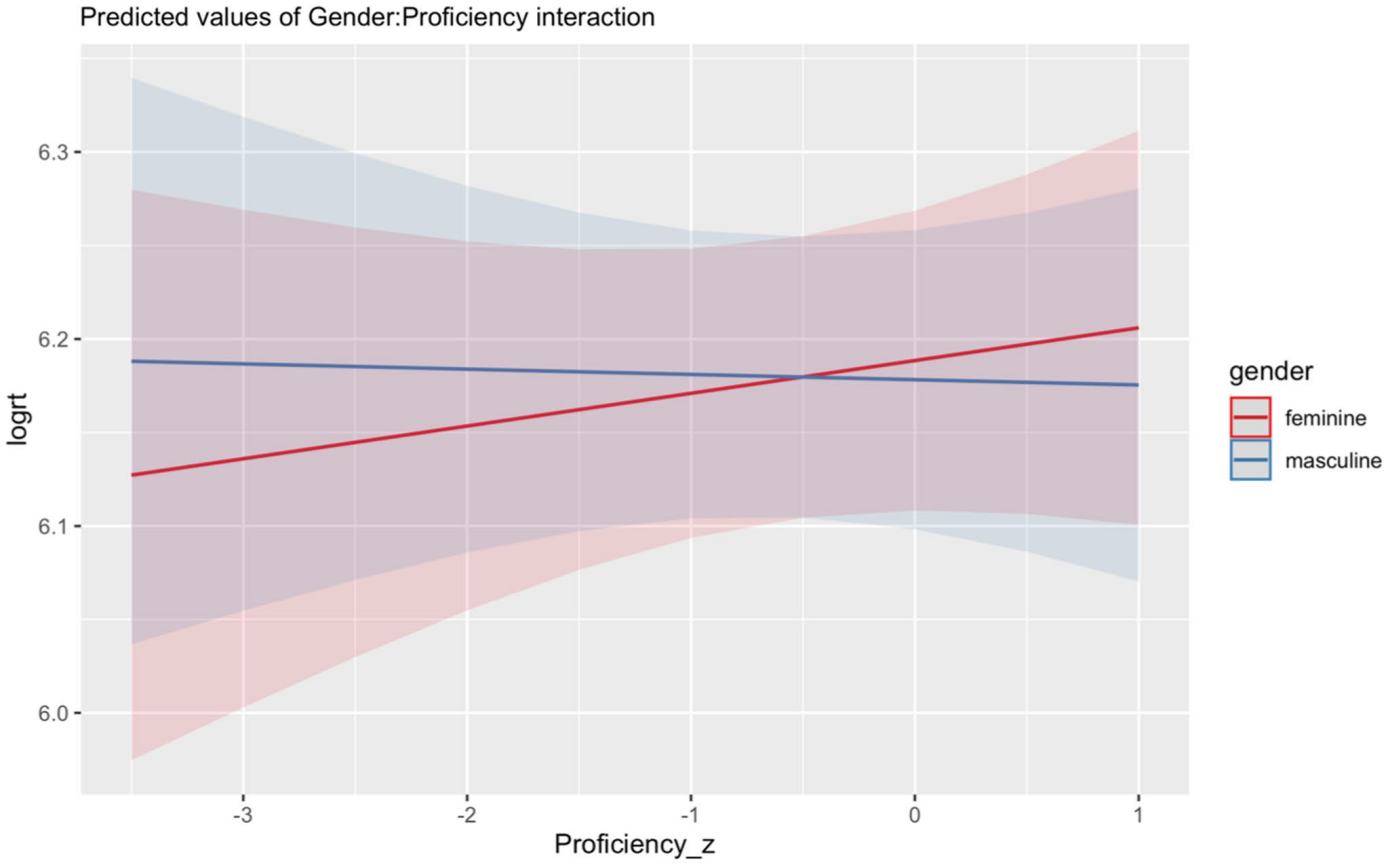
Illustration of the interaction between *Gender* (feminine, masculine) and Proficiency scores for Heritage Speakers.

To investigate whether proficiency and HL use may have affected RTs in HSs, we fitted a model to the heritage group data for Region 6 and Region 7. In Region 6, there were no significant interactions for any of the predictors (*p*’s > 0.05). In Region 7, the model revealed a main effect of *Grammaticality* (Chisq = 6.20, *p* = 0.013), indicating that HSs were sensitive to ungrammaticalities which were reflected in shorter RTs in the grammatical compared to the ungrammatical condition. The significant interaction between *Grammaticality*:*Gender* (Chisq = 4.81, *p* = 0.028) confirmed that in the grammatical conditions, there was no difference in RTs between feminine and masculine (*β* = 0.006, *SE* = 0.020, *z* = 0.334, *p* = 0.987); however, in the ungrammatical conditions, HSs showed shorter RTs for ungrammatical feminine as compared to ungrammatical masculine (*β* = -0.060, *SE* = 0.020, *z* = -3.039, *p* = 0.013). The significant interaction between *Gender*:*Proficiency* (Chisq = 5.21, *p* = 0.023) indicates that HSs had shorter RTs for sentences with masculine compared to feminine nouns as their proficiency increased (Figure 4).

In summarizing, the online RT data revealed that overall HSs had longer RTs compared to homeland speakers. In the critical and post-critical regions, both groups showed longer RTs for ungrammatical sentences; however, HSs showed significantly longer RTs for feature clash errors when ungrammatical sentences were realized on feminine marked adjectives compared to default errors realized on masculine unmarked adjectives. Finally, proficiency was the only extra-linguistic predictor of HSs’ RT performance, while other extra-linguistic factors were not significant.

### Grammaticality judgement task

**Figure 5:**
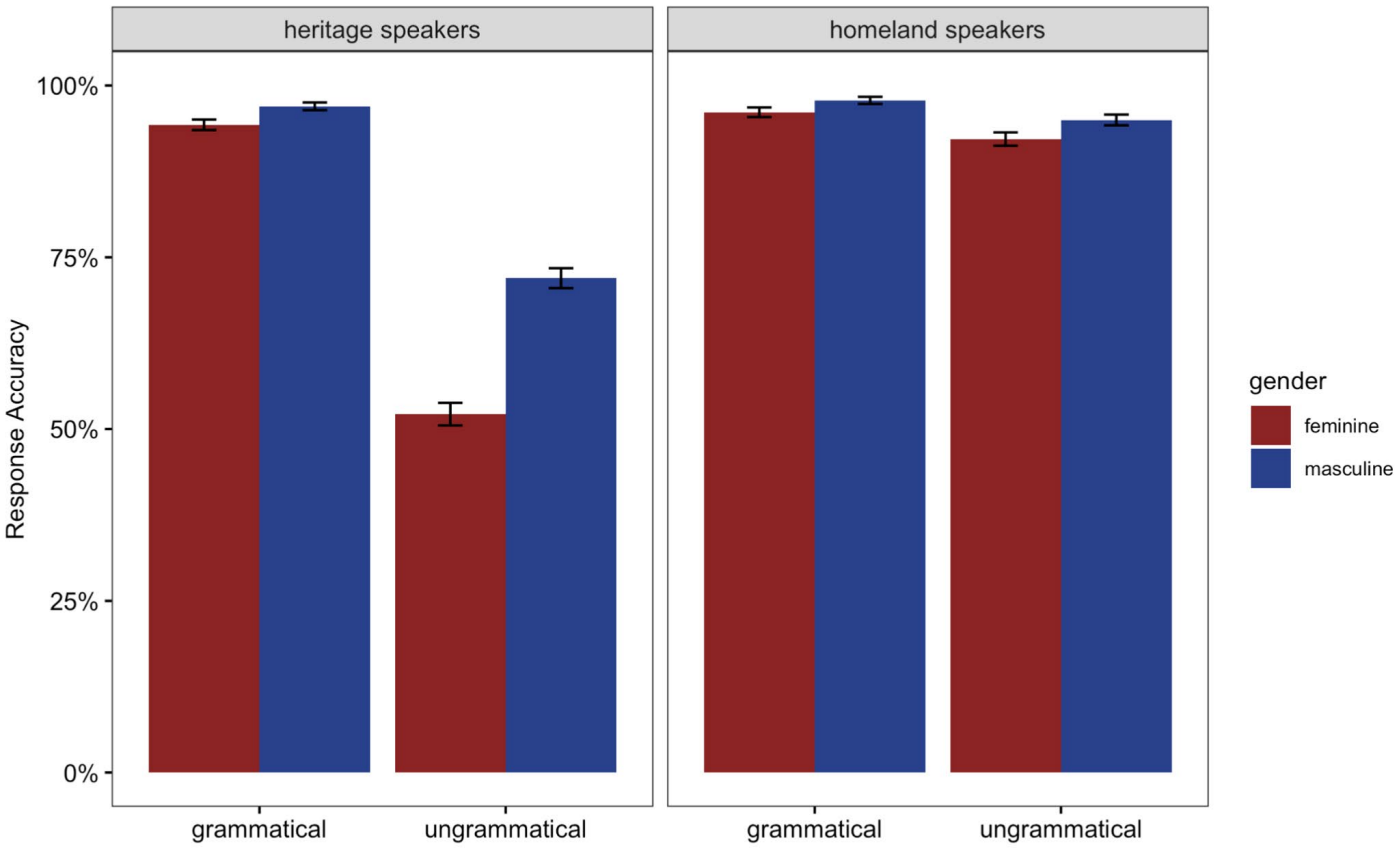
Mean response accuracy in percentage for the grammatical and ungrammatical conditions per group in the Grammaticality Judgment Task (GJT). The bars represent the standard error to the mean.

The results for the GJT are presented in Figure 5 and Table 2. Homeland speakers performed above 90% in all conditions. HSs’ performance was above 90% in the grammatical conditions; however, HSs were less likely to judge accurately ungrammatical feminine sentences (default errors) where the violation was realized on masculine unmarked adjectives (52%) compared to ungrammatical masculine sentences (feature clash error) where the violation was realized on feminine marked adjectives (72%).

**Table 2:** Mean accuracy scores (%) and SDs per condition for HSs and homeland speakers in the GJT.

	Heritage speakers	Homeland speakers
**Condition**	M (*SD*)	M (*SD*)
*Grammatical feminine*	94 (*0.23*)	96 (*0.19*)
*Grammatical masculine*	97 (*0.17*)	98 (*0.14*)
*Ungrammatical feminine*	52 (*0.50*)	92 (*0.27*)
*Ungrammatical masculine*	72 (*0.45*)	95 (*0.22*)

The model revealed a significant main effect of *Group* (Chisq = 31.91, *p* < 0.001) showing that HSs were overall significantly less accurate as compared to homeland speakers. The significant effect of *Grammaticality* (Chisq = 62.99, *p* < 0.001) shows that both groups were overall significantly more accurate with the grammatical conditions as compared to the ungrammatical ones. The effect of *Gender* (Chisq = 21.45, *p* < 0.001) was also significant and indicates that both HSs and homeland speakers were less accurate with sentences containing feminine marked nouns. The significant interaction between *Group*:*Grammaticality* (Chisq = 24.12, *p* < 0.001) and subsequent *post-hoc* pairwise comparisons showed that in the grammatical conditions, there was no difference in accuracy between HSs and homeland speakers (*β* = -0.396, *SE* = 0.304, *z* = -1.305, *p* = 0.192). However, in the ungrammatical conditions, HSs performed significantly less accurately than homeland speakers (*β* = -2.585, *SE* = 0.337, *z* = -7.668, *p* < 0.001). There was no significant three-way interaction between *Group:Grammaticality:Gender* (Chisq = 0.97, *p* = 0.325). However, since this was our *a priori* comparison, we run *post-hoc* pairwise comparisons that showed that in the grammatical conditions, the difference in accuracy between feminine and masculine nouns was not different between HSs and homeland speakers (*β* = -0.112, *SE* = 0.439, *z* = -0.256, *p* = 0.798). In the ungrammatical conditions, however, the difference in accuracy between feminine and masculine nouns was statistically different between HSs and homeland speakers (*β* = -0.603, *SE* = 0.297, *z* = -2.030, *p* = 0.042), indicating that HSs were less accurate with the feminine nouns (where the ungrammaticality was caused by a masculine unmarked adjective—default error) compared to the masculine ones (where the ungrammaticality was caused by a feminine marked adjective—feature clash error).

**Figure 6:**
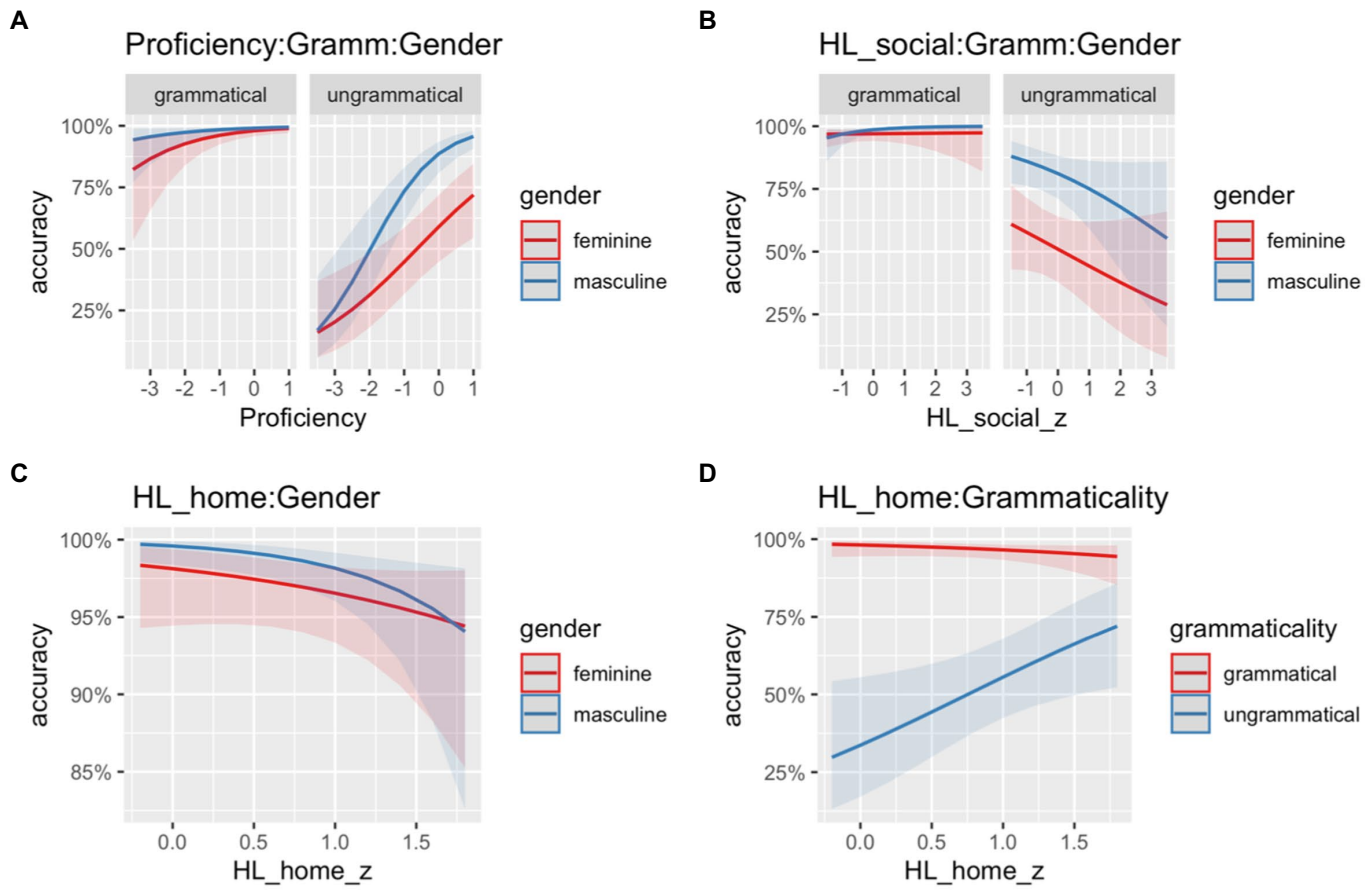
**(A)** Illustration of the three-way interaction between *Proficiency*, *Grammaticality* (grammatical, ungrammatical), and *Gender* (feminine, masculine); **(B)** Illustration of the three-way interaction between *heritage exposure outside of home context* (HL_social), *Grammaticality* (grammatical, ungrammatical), and *Gender* (feminine, masculine); **(C)** Illustration of the two-way interaction between *heritage exposure at home* (HL_home) and *Gender* (feminine, masculine); and **(D)** Illustration of the two-way interaction between *heritage exposure at home* (HL_home) and *Grammaticality* (grammatical, ungrammatical).

The model fitted to the heritage group data revealed a main effect of *Grammaticality* (Chisq = 227.10, *p* < 0.001), indicating that HSs were more accurate with grammatical conditions compared to ungrammatical ones. The main effect of *Gender* (Chisq = 26.43, *p* < 0.001) indicates that HSs were less accurate with sentences containing feminine marked nouns. Furthermore, the model revealed that *Proficiency* (Chisq = 15.62, *p* < 0.001) was a significant predictor of accuracy in the GJT: the higher the score in the vocabulary test, the better the performance in the task. There was also a significant two-way interaction between *Gender* and *HL_home* (Chisq = 4.34, *p* = 0.037), as well as *Grammaticality* and *HL_home* (Chisq = 28.20, *p* < 0.001) as illustrated in Figures 6C,D. These interactions suggest that with more exposure to the HL at home, the smaller the differences are in accuracy between feminine and masculine nouns (Figure 6C) as well as grammatical and ungrammatical items (Figure 6D). There was also a significant three-way interaction between *Grammaticality*, *Gender*, and *HL_social* (Chisq = 4.35, *p* = 0.037), and *Grammaticality*, *Gender*, and *Proficiency* (Chisq = 4.51, *p* = 0.034), as illustrated in Figures 6A,B. It appears to be the case that with increasing proficiency, HSs are more sensitive to the distinction between feature clash vs. default error patterns, as shown in Figure 6A (i.e., the interaction is mainly driven from the difference between feminine and masculine nouns in the ungrammatical condition). Moreover, more exposure to the HL in social contexts seems to modulate the difference in accuracy between feminine and masculine nouns in the grammatical condition, suggesting that with more exposure, HSs have higher accuracy for masculine nouns.

To summarize, the accuracy data show that both groups were more accurate with grammatical conditions as compared to ungrammatical ones. Furthermore, both HSs and homeland speakers were more accurate with sentences containing masculine unmarked nouns as compared to sentences with feminine marked nouns. There was no difference between groups in the grammatical conditions; however, in the ungrammatical conditions, HSs were more accurate in detecting violations realized on feminine marked adjectives (feature clash errors) compared to violations realized on the masculine unmarked adjectives (default errors). Finally, HSs’ proficiency as well language use was predictors of accuracy, whereas the type of bilingualism was not.

## Discussion

The present study investigated whether and how morphological markedness influences gender agreement processing in Italian, comparing how this manifests in both native homeland and HSs. Focusing on the HSs, we examined whether RTs and accuracy were calibrated to proficiency, individual HL use, and/or task modality (online vs. offline). To this aim, participants completed an online SPRT and an offline GJT where markedness of the trigger nouns was systematically manipulated, isolating the potentially unique contribution of markedness to noun-adjective agreement resolution.

To summarize the entirety of the data, RTs revealed that overall HSs were reading at a slower speed compared to homeland speakers. This is not surprising, considering that HSs have less experience with reading in Italian and lower proficiency. In the critical and post-critical regions, both groups showed overall longer RTs during processing of ungrammaticality indicating that the method was successful. In the grammatical conditions, we found no difference in RTs between feminine and masculine nouns between HSs and homeland speakers, whereas in the ungrammatical conditions, only the HSs showed signs that markedness played a specific role in processing agreement. Interestingly, HSs displayed significantly longer RTs when the ungrammatical sentences were realized on feminine marked adjectives (feature clash error) compared to masculine unmarked adjectives (default error), this was not true of the homeland speakers.

The offline data show that both groups were more accurate with grammatical conditions as compared to ungrammatical conditions. Markedness mattered here for both, given higher accuracy with sentences containing masculine unmarked nouns as compared to feminine marked nouns. In the grammatical conditions, there was no difference between the groups; however, in the ungrammatical conditions while the homeland speakers displayed very high (above 90%) accuracy, HSs were less accurate in detecting violations realized on the masculine unmarked adjectives (ungrammatical feminine—default error) compared to violations realized on feminine marked adjectives (ungrammatical masculine—feature clash error)–the same condition for which they were slower in RTs. That is, there seems to be an apparent *speed-accuracy tradeoff* for HSs conditioned by markedness, a point to which we return below. In both tasks, proficiency was a significant extra-linguistic predictor–speed and accuracy increased as HSs’ proficiency increased–while HL use only mattered for accuracy. In the remainder of this section, we discuss how these results fit into the larger context of the relevant literature more generally.

With respect to our first research question concerning whether or not there is an effect of markedness at all, we see clear evidence that markedness matters for HSs and for homeland natives; however, this plays out differently depending on the group and the task. The offline evidence from both groups converges on the fact that masculine is the default gender in Italian (D'Achille, 2003; Corbett and Fraser, 2011). In this respect, the overall present data are in line with other studies reporting higher accuracy with masculine nouns compared to feminine ones for HSs of Romance languages (i.e., Montrul et al., 2008; Alarcón, 2011; Bianchi, 2013; Kupisch et al., 2013; Irizarri van Suchtelen, 2016; Hur et al., 2020) as well as for homeland native speakers and L2 speakers (for Spanish: McCarthy, 2008; for Italian and French: Vigliocco and Franck, 1999, 2001).

However, as it pertains to gender agreement violations—how markedness affects the processing of ungrammaticality—we see a difference between the two groups that at first glance might seem counterintuitive. The results showed that HSs were more sensitive to ungrammaticality when realized on feminine marked adjectives, that is, an error denoting a feature clash running counter to morphological markedness. One might have expected such an error to be as salient, potentially more so, for homeland Italian speakers considering that a feature clash error is argued to be more costly for processing in general and/or attributable to the status of gender specification. Neuroimaging studies, for example Alemán Bañón and Rothman (2016), have shown that homeland natives of Spanish should increase amplitude for these types of errors relative to default agreement ones. Our results are also highly reminiscent of what Fuchs et al. (2015) showed for Spanish homeland speakers, leading them to argue that such patterns provide empirical evidence for the position that masculine in Romance (at least in Spanish) should be evaluated as the absence of a gender specification. Indeed, our results for the HSs speak to the same argumentation for Italian gender, but the fact that the homeland Italian speakers do not align with the Spanish ones in Fuchs et al. (2015) to the same degree leaves us a bit reluctant to make the same conclusions definitively. As alluded to above, our Italian homeland speakers were quite fast in reading these sentences. While the homeland speakers do show a distinction in overall reading time between grammatical and ungrammatical agreement sentences, the lack of markedness might be attributable to the granularity of what a RT study might be able to reveal as compared to an EEG study as in Alemán Bañón and Rothman (2016) or somehow what could be shown in an auditory acceptability task as in Fuchs and colleagues. Considering the fact that the homeland speakers had above 90% accuracy in both grammatical and ungrammatical conditions and very fast in reading no matter the sentence type, it could be the case that this method is simply unable to tease out any fine-grained effects that markedness might have otherwise conditioned. The case of HSs is distinct precisely because they are not (as) quick readers of Italian and they are not universally at ceiling with all sentence types. Given that they are slower overall and not at ceiling with ungrammatical sentences, an online/RT method had a better chance at revealing an underlying effect for markedness *a priori*. As we noted above, there was a tradeoff between accuracy and speed in the HSs only; the slower the reading, the greater the accuracy with HSs. This tradeoff afforded the HSs the opportunity to process what they were reading, and thus, the effect of markedness had time to reveal itself. Alternatively, or perhaps working in tandem, the fact that HSs might rely more on morphological defaults generally could be significant here. In the present case, a heightened reliance of defaults—a tendency that has been reported also for monolingual (i.e., Pérez-Pereira, 1991) and bilingual (i.e., Eichler et al., 2012) children as well as for L2 learners (i.e., Franceschina, 2001; McCarthy, 2008)—might make feature clash errors even more disruptive for HS processing than for homeland speakers. Coupling a potential HS heightened sensitivity to defaults with the slower overall reading times of HSs and lack of ceiling effects with ungrammatical agreement could have all combined to give rise to the differences we noted in the groups.

Our second research question explored the effects of proficiency and extra-linguistic factors on the processing of gender agreement in HSs. We found that proficiency was a significant extra-linguistic predictor of accuracy and RTs. This is in line with previous studies (i.e., Bianchi, 2013; Kupisch et al., 2013) reporting accuracy for HSs to be modulated by proficiency in the HL. Specifically, the effect of markedness is modulated by proficiency, as shown in Figure 6A, such that the higher the proficiency, the stronger the effect of markedness; thus, HSs are more sensitive to the distinction between feature clash vs. default error patterns. It is worth noting that the proficiency test we used was a measure of lexical knowledge, which is only one dimension of proficiency. However, lexical proficiency has been shown to be a reliable measure for overall language proficiency (i.e., Alderson, 2005) and positive correlations have been found between HSs’ lexical knowledge and overall HL proficiency (i.e., Daller et al., 2003; Lloyd-Smith et al., 2019).

Based on previous studies on adult HSs, we were expecting an effect of HL use (i.e., Bianchi, 2013) and AoO of bilingualism (i.e., Montrul, 2008; Keating, 2022); however, we did not find any effect of AoO, whereas HL use affected only accuracy. Specifically, our results showed that the more use and exposure to the HL at home and in social contexts, the smaller the differences are in accuracy between feminine and masculine nouns. This finding suggests that gender is sensitive to input effects. Our results, supported by HS performance in the GJT, are in line with Bianchi (2013), showing that HL use in adulthood plays a major role during processing of gender agreement, regardless of AoO of bilingualism. Gender in Italian is acquired early (Chini, 1995; Belletti and Guasti, 2015) and it is a considerably transparent system (Kupisch et al., 2002; Padovani and Cacciari, 2003); thus, it is less challenging to acquire and maintain gender in Italian. The reader may recall that in our study, we tested adult HSs who have relatively high proficiency, perhaps significantly so in this European context as compared to studies in other contexts, such as North America, where gender in seemingly linguistically comparable HLs (e.g., Spanish) has been depicted as being vulnerable.

Finally, our third research question investigated whether HSs’ use of markedness was affected by task modality (online vs. offline). We hypothesized to find a markedness effect in both tasks; however, we left open the possibility that the degree of the effect would differ between the tasks. Our results showed that HSs were sensitive to markedness in both online and offline comprehension, however in a different way, indicating as discussed above a *speed-accuracy tradeoff* effect. In any task that requires control over both accuracy and responses, participants can optimize either speed or accuracy, or a compromise between the two. Such a compromise leads to increasing speed at the cost of accuracy or increasing accuracy at the cost of speed. This trade-off is widely attested as evidence for development in cognitive control–as children get older and are faced with more challenging tasks, they tend to preserve their accuracy by sacrificing their speed (Davidson et al., 2006; Best et al., 2011). Furthermore, Struys et al. (2018) found that bilinguals tend to show a stronger relationship between speed and accuracy in their performance on cognitive tasks than the monolinguals, suggesting that bilinguals tend to rely more on this optimization strategy to boost their performance. Indeed, bilinguals develop specific strategies to resolve various linguistic conflicts (Muysken, 2013), and our results also corroborate this by demonstrating that HSs chose a strategy to boost accuracy at cost of slower response times on detecting violations conditioned by markedness–a linguistic feature that has been shown to be costly to process for bilinguals.

To conclude, our results indicate that both homeland speakers and HSs access and make use of markedness information during processing of agreement in online and offline sentence comprehension. Most importantly, only HSs showed greater sensitivity to feature clash errors which resulted in slower RTs and higher accuracy on ungrammaticality realized on feminine marked adjectives. Future studies examining gender processing in HSs should consider the effect of markedness both at the level of gender feature on the noun (marked vs. unmarked gender) and errors that involve mismatching marked features (feature clash errors vs. default errors).

## Data availability statement

The raw data supporting the conclusions of this article will be made available by the authors, without undue reservation, to any qualified researcher.

## Ethics statement

This study was reviewed by the University of Konstanz Research Ethics Committee and was given a favorable ethical opinion for conduct (IRB 29/2019). The participants provided their written informed consent to participate in this study.

## Author contributions

GP, TM, and JR designed and conducted the study. GP and MK analyzed the data. GP, MK, JR, and TM wrote the paper. JR and TM commented and revised the paper. All authors contributed to the article and approved the submitted version.

## Funding

This article was supported by generous funding to GP by the European Union’s Horizon 2020 research and innovation program under the Marie Skłodowska Curie grant agreement No. 765556. JR was funded by the Tromsø Forskningsstiftelse (Tromsø Research Foundation) starting grant No. A43484 and the Heritage-bilingual Linguistic Proficiency in their Native Grammar (HeLPiNG; 2019–2023).

## Conflict of interest

The authors declare that the research was conducted in the absence of any commercial or financial relationships that could be construed as a potential conflict of interest.

## Publisher’s note

All claims expressed in this article are solely those of the authors and do not necessarily represent those of their affiliated organizations, or those of the publisher, the editors and the reviewers. Any product that may be evaluated in this article, or claim that may be made by its manufacturer, is not guaranteed or endorsed by the publisher.
