# Supplementary material for: Effects of markedness in gender processing in Italian as a heritage language: A speed accuracy tradeoff
Source: Front Psychol. 2022 Oct 12;13:965885. doi: 10.3389/fpsyg.2022.965885 (PMC9596986; doi:10.3389/fpsyg.2022.965885)
Supplement: Supplementary file 1 [file Data_Sheet_1.docx]

**Supplementary Materials.**

**Table S1.** Table summarizing the indices obtained in the background questionnaire.

|  | **heritage speakers** | **homeland speakers** |
| --- | --- | --- |
|  |  |  |
|  | **M(SD)** | **M(SD)** |
|  | **Range** | **Range** |
|  |  |  |
| **Age** | 28(6.20) | 26(3.99) |
|  | 18-41 | 18-39 |
|  |  |  |
| **AoO German in years** |  |  |
| **Simultaneous (N=33)** | 0 | - |
| **Sequential (N=21)** | 1.5(1.97) | - |
|  | 3-6 | - |
|  |  |  |
| **Self-rated proficiency^1^ - range(1-40)** | 32(5.26) | 39(1.62) |
|  | 20-40 | 33-40 |
|  |  |  |
| **Speaking (max. 10)** | 8(1.40) | 10(0.66) |
|  | 4-10 | 7-10 |
|  |  |  |
| **Listening (max. 10)** | 9(1.19) | 10(0.38) |
|  | 5-10 | 8-10 |
|  |  |  |
| **Reading (max. 10)** | 8(1.71) | 10(0.33) |
|  | 4-10 | 9-10 |
|  |  |  |
| **Writing (max. 10)** | 7(1.71) | 10(0.54) |
|  | 4-10 | 8-10 |
|  |  |  |
| **Dialang score (max. score 75)** | 60(6.49) | 70(2.33) |
|  | 44-70 | 66-75 |
|  |  |  |
| **Language before 6 years old^2^** | 4(1.05) | 5(0.96) |
|  | 2-5 | 0-5 * |
|  |  |  |
| **Language after 6 years old^3^** | 4(1.05) | 5(0.99) |
|  | 1-5 | 0-5 * |
|  |  |  |
| **Language used now^4^** | 3(0.99) | 4(1.09) |
|  | 1-5 | 0-5 * |
|  |  |  |
| **Language used during school and university^5^** | 2(0.92) | 4(0.71) |
|  | 1-5 | 2-5 |
|  |  |  |
| **Italian language courses^6^**  **(N=39 participants reported to have done Italian courses)** |  |  |
| **Number of courses** | 3(1.85) | - |
|  | 1-5 | - |
| **Length of the longest course** | 4(1.41) | - |
|  | 1-5 | - |
| **Frequency of the longest course** | 2(1.33) | - |
|  | 1-5 | - |
| **Tot. number of courses in years** | 3(1.46) | - |
|  | 1-5 | - |
|  |  |  |
| **Current conversation habit^7^** | 4(1.13) | 4(0.97) |
|  | 3-6 | 3-6 |
|  |  |  |
| **Conversation habit in the past^8^** | 3(1.18) | 4(0.76) |
|  | 1-5 | 3-6 |
|  |  |  |
| **HL/Italian use in daily life^9^** | 3(0.56) | 4(0.78) |
|  | 2-4 | 1-5 * |
|  |  |  |
| **Quality of HL^10^** | 2(0.62) | 4(0.62) |
|  | 1-4 | 3-5 |
|  |  |  |
| **HL use in the home (LSBQ)^11^** | 10.85(3.93) | -3.11(5.13) ** |
|  | 3.17-18.94 | -9.86-9.92 |
|  |  |  |
| **HL use in the society (LSBQ)^12^** | 13.97(10.42) | 11.62(11.88) ** |
|  | -1.69-47.97 | -6.94-44.69 |
|  |  |  |

* Some Homeland Italian speakers considered their dialect as an “other language”.

** The Homeland Italian speakers were not monolingual (although they all grew up monolingually in Italy), thus in the LSBQ they reported the use of a L2 compared to Italian (N=30 participants reported English as L2; N=4 participants reported Spanish as L2; N=3 participants reported French as L2; N=2 participants reported German as L2; N=1 participants reported Russian as L2).

^1^Self-rated proficiency: 10 points for each language skill (speaking; listening; reading; writing).

^2^Language used at home before 6 years old: other language=0; only German=1; mainly German=2; half German/half Italian=3; mainly Italian=4; only Italian=5.

^3^Language used at home after 6 years old: other language=0; only German=1; mainly German=2; half German/half Italian=3; mainly Italian=4; only Italian=5.

^4^Language used at home now: other language=0; only German=1; mainly German=2; half German/half Italian=3; mainly Italian=4; only Italian=5.

^5^Language used during school and university: Language used in ‘elementary school’, middle school’, high school’, ‘Bachelor’, ‘Master’, PhD’; for each level only German=1; mainly German=2; half German/half Italian=3; mainly Italian=4; only Italian=5.

^6^ Italian language courses:

Number of courses: 1= 1 course; 2= 2 courses; 3= 3 courses; 4= 4 courses; 5= 5 or more courses.

Length of the longest course: 1= 1 month or less; 2= more than 1 month; 3= more than 6 months; 4= more than 1 year; 5= more than 3 years.

Frequency of the longest course: 1 = 1 course per week; 2 = 2 courses per week; 3 = 3 courses per week; 4 = 4 courses per week; 5 = 5 courses per week.

Tot. number of courses in years: 1= one year or less; 2= 2 or 3 years; 3= 4-7 years; 4= 8-11 years; 5= 12 or more years.

^7^Currrent conversation habit: No. of people Italian is spoken with – currently: 0 person=1; 1 person=2; 2-5 people=3; 6-10 people=4; 11-20 people=5; 20+ people=6.

^8^Conversation habit in the past: No. of people Italian was spoken with – in the past: 1 person= 1; 2-5 people=2; 6-10 people=3; 11-20 people=4; 20+ people=5.

^9^HL use in daily life: Language used with mother, father, siblings, grandparents, partner, friends, flat-mates, colleagues; only German=1; mainly German=2; half German/half Italian=3; mainly Italian=4; only Italian=5.

^10^Quality of HL: Language used at work, at the university, for social activities, for writing emails and texts, for watching movies and listening to music, for internet, for expressing emotions, for calculating, for reading; only German=1; mainly German=2; half German/half Italian=3; mainly Italian=4; only Italian=5.

From the LSBQ we used two factor scores:

^11^HL use in the home (possible range: –13.9 to 24.163, the higher the score, the more the HL is used in home settings)

^12^HL use in society (possible range: –7.5 to 80.304, the higher the score, the more frequently the HL is used in social settings)

For both factors, a higher score indicates more use of the HL (Italian)/L2 for homeland speakers, while a lower score indicates more use of the ML (German)/ Italian for homeland speakers.

**1. List of critical nouns used in the tasks.**

| **Feminine nouns** | **Translation** | **Ending** | **Congruency** |
| --- | --- | --- | --- |
| FALCE | *sickle* | opaque | congruent |
| FONTE | *source* | opaque | congruent |
| INDAGINE | *investigation* | opaque | congruent |
| NOCE | *walnut* | opaque | congruent |
| PARETE | *wall* | opaque | congruent |
| PARTE | *part/role* | opaque | congruent |
| RONDINE | *swallow* | opaque | congruent |
| SERPE | *snake* | opaque | congruent |
| VITE | *screw* | opaque | congruent |
| VOCE | *voice* | opaque | congruent |
| CHIAVE | *key* | opaque | incongruent |
| FEDE | *wedding ring* | opaque | incongruent |
| FRASE | *sentence* | opaque | incongruent |
| LEPRE | *hare* | opaque | incongruent |
| LITE | *fight* | opaque | incongruent |
| PALUDE | *swamp* | opaque | incongruent |
| PATENTE | *driving license* | opaque | incongruent |
| SEDE | *location* | opaque | incongruent |
| TORRE | *tower* | opaque | incongruent |
| VOLPE | *fox* | opaque | incongruent |
| ABITUDINE | *habit* | transparent | congruent |
| LAVATRICE | *washing machine* | transparent | congruent |
| CICATRICE | *scar* | transparent | congruent |
| ALICE | *anchovy* | transparent | congruent |
| OCCASIONE | *opportunity* | transparent | congruent |
| OPINIONE | *opinion* | transparent | congruent |
| QUESTIONE | *issue* | transparent | congruent |
| RIUNIONE | *meeting* | transparent | congruent |
| SOLUZIONE | *solution* | transparent | congruent |
| RADICE | *root* | transparent | congruent |
| VERNICE | *paint* | transparent | incongruent |
| FUNZIONE | *religious function* | transparent | incongruent |
| CORNICE | *frame* | transparent | incongruent |
| FRODE | *fraud* | transparent | incongruent |
| RELAZIONE | *report* | transparent | incongruent |
| STAMPANTE | *printer* | transparent | incongruent |
| LEZIONE | *lesson* | transparent | incongruent |
| STAZIONE | *station* | transparent | incongruent |

| **Masculine nouns** | **Translation** | **Ending** | **Congruency** |
| --- | --- | --- | --- |
| CANE | *dog* | opaque | congruent |
| TORRENTE | *stream* | opaque | congruent |
| DENTE | *tooth* | opaque | congruent |
| DOLCE | *dessert* | opaque | congruent |
| FIUME | *river* | opaque | congruent |
| MESE | *month* | opaque | congruent |
| MONTE | *mountain* | opaque | congruent |
| PESCE | *fish* | opaque | congruent |
| FULMINE | *lightning* | opaque | congruent |
| PETTINE | *comb* | opaque | congruent |
| ABETE | *fir tree* | opaque | incongruent |
| BAULE | *chest* | opaque | incongruent |
| GIRASOLE | *sunflower* | opaque | incongruent |
| ENTE | *company* | opaque | incongruent |
| GREMBIULE | *apron* | opaque | incongruent |
| FUCILE | *rifle* | opaque | incongruent |
| PARERE | *opinion* | opaque | incongruent |
| PONTE | *bridge* | opaque | incongruent |
| SERPENTE | *snake* | opaque | incongruent |
| BASTONE | *cane* | transparent | congruent |
| POSACENERE | *ashtray* | transparent | congruent |
| ERRORE | *mistake* | transparent | congruent |
| POLPETTONE | *meatloaf* | transparent | congruent |
| INCIDENTE | *accident* | transparent | congruent |
| MAGLIONE | *sweater* | transparent | congruent |
| PALLONE | *football* | transparent | congruent |
| RIGORE | *penalty shot* | transparent | congruent |
| RUMORE | *noise* | transparent | congruent |
| CACCIAVITE | *screwdriver* | transparent | congruent |
| CANTIERE | *construction site* | transparent | incongruent |
| COLORE | *color* | transparent | incongruent |
| FAVORE | *favor* | transparent | incongruent |
| FIORE | *flower* | transparent | incongruent |
| GIACCONE | *winter jacket* | transparent | incongruent |
| LEGAME | *bond* | transparent | incongruent |
| INGREDIENTE | *ingredient* | transparent | incongruent |
| TEGAME | *pan* | transparent | incongruent |
| ESAME | *exam* | transparent | incongruent |
| SAPONE | *soap* | transparent | incongruent |

**TableS2**. Percentage of removed data and final data pool per task per group.

|  | **Heritage** | | | **Homeland** | | |
| --- | --- | --- | --- | --- | --- | --- |
|  | **SPRT** | **GJT** | **GAT** | **SPRT** | **GJT** | **GAT** |
| initial data pool | 33.603 data points | 4.314  data points | 4.158  data points | 26.720 data points | 3.199  data points | 3.080  data points |
| trimming: unknown words | 6.5% | 6.4% | 6.6% | -- | -- | -- |
| trimming: GAT | 6.8% | 6.8% | -- | 3.1% | 3.1% | -- |
| trimming: extreme values | 1.1% | -- | -- | 2.2% | -- | -- |
| trimming: outliers | 2.2% | -- | -- | 3.6% | -- | -- |
| final data pool | 28.718 data points | 3.761  data points | 3.883  data points | 23.433 data points | 3.098  data points | 3.080  data points |

**2. Model specifications for the statistical analyses in section 4.1 (SPRT)**

**Region 5: Pre-critical region (Noun)**

**Between groups**

Model:

*(log)rt ~ grammaticality + group + gender + grammaticality:group + grammaticality:gender + group:gender + grammaticality:group:gender + (grammaticality+gender| subject) + (grammaticality+gender| item)*

**Table 2.1** Model output.

| **Model output for RTs in the SPRT - Region 5 noun** | | | | |  |
| --- | --- | --- | --- | --- | --- |
| *Effect* | *df* | *Chisq* | *p* |  |  |
| grammaticality[grammatical] | 1 | .42 | .519 |  |  |
| group[heritage] | 1 | 19.74 | **<.001** | ******* |  |
| gender[feminine] | 1 | .33 | .567 |  |  |
| grammaticality[grammatical]:group[heritage] | 1 | .04 | .852 |  |  |
| grammaticality[grammatical]:gender[feminine] | 1 | .00 | .995 |  |  |
| group[heritage]:gender[feminine] | 1 | 1.29 | .257 |  |  |
| grammaticality[grammatical]:group[heritage]:gender[feminine] | 1 | .32 | .572 |  |  |
| Signif. codes: 0 ‘***’, 0.001 ‘**’, 0.01 ‘*’, 0.05 ‘+’, 0.1 ‘ ’ 1 |  |  |  |  |  |

**Heritage group**

Model:

*(log)rt ~ grammaticality + gender + dialang_z + bilingualism + HL_home + HL_social + grammaticality:gender + grammaticality:dialang_z + grammaticality:bilingualism + grammaticality:HL_home + grammaticality:HL_social + gender:dialang_z + gender:bilingualism + gender:HL_home + gender:HL_social + grammaticality:gender:dialang_z + grammaticality:gender:bilingualism + grammaticality:gender:HL_home + grammaticality:gender:HL_social + (1| subject) + (1| item)*

**Table 2.3** Model output.

| **Model output for RTs in the SPRT - Region 5 noun - Heritage** | | | | |
| --- | --- | --- | --- | --- |
| *Effect* | *df* | *Chisq* | *p* |  |
| grammaticality[grammatical] | 1 | .26 | .611 |  |
| gender[feminine] | 1 | .19 | .664 |  |
| dialang_z | 1 | 3.00 | .083 | + |
| bilingualism | 1 | 1.23 | .267 |  |
| HL_home_z | 1 | 2.28 | .131 |  |
| HL_social_z | 1 | .57 | .450 |  |
| grammaticality[grammatical]:gender[feminine] | 1 | .17 | .680 |  |
| grammaticality[grammatical]:dialang_z | 1 | .35 | .556 |  |
| grammaticality[grammatical]:bilingualism | 1 | 2.81 | .094 | + |
| grammaticality[grammatical]:HL_home_z | 1 | .06 | .812 |  |
| grammaticality[grammatical]:HL_social_z | 1 | .56 | .456 |  |
| gender[feminine]:dialang_z | 1 | .68 | .409 |  |
| gender[feminine]:bilingualism | 1 | .00 | .988 |  |
| gender[feminine]:HL_home_z | 1 | 2.24 | .135 |  |
| gender[feminine]:HL_social_z | 1 | .84 | .360 |  |
| grammaticality[grammatical]:gender[feminine]:dialang_z | 1 | 1.47 | .226 |  |
| grammaticality[grammatical]:gender[feminine]:bilingualism | 1 | .15 | .700 |  |
| grammaticality[grammatical]:gender[feminine]:HL_home_z | 1 | .03 | .871 |  |
| grammaticality[grammatical]:gender[feminine]:HL_social_z | 1 | .37 | .541 |  |
| Signif. codes: 0 ‘***’, 0.001 ‘**’, 0.01 ‘*’, 0.05 ‘+’, 0.1 ‘ ’ |  |  |  |  |

**Region 6: Critical region (Adjective)**

**Between groups**

Model:

*(log)rt ~ grammaticality + group + gender + grammaticality:group + grammaticality:gender + group:gender + grammaticality:group:gender + (grammaticality+gender| subject) + (grammaticality+gender| item)*

**Table 2.2** Model output.

| **Model output for RTs in the SPRT - Region 6 adjective** | | | | |  |
| --- | --- | --- | --- | --- | --- |
| *Effect* | *df* | *Chisq* | *p* |  |  |
| grammaticality[grammatical] | 1 | 5.76 | **.016** | ***** |  |
| group[heritage] | 1 | 27.67 | **<.001** | ******* |  |
| gender[feminine] | 1 | .10 | .746 |  |  |
| grammaticality[grammatical]:group[heritage] | 1 | .48 | **.488** |  |  |
| grammaticality[grammatical]:gender[feminine] | 1 | 1.80 | **.180** |  |  |
| group[heritage]:gender[feminine] | 1 | 5.22 | **.022** | ***** |  |
| grammaticality[grammatical]:group[heritage]:gender[feminine] | 1 | 2.78 | **.096** | **+** |  |
| Signif. codes: 0 ‘***’, 0.001 ‘**’, 0.01 ‘*’, 0.05 ‘+’, 0.1 ‘ ’ 1 |  |  |  |  |  |

**Heritage group**

Model:

*(log)rt ~ grammaticality + gender + proficiency + bilingualism + HL_home + HL_social + grammaticality:gender + grammaticality:proficiency + grammaticality:bilingualism + grammaticality:HL_home + grammaticality:HL_social + gender:proficiency + gender:bilingualism + gender:HL_home + gender:HL_social + grammaticality:gender:proficiency + grammaticality:gender:bilingualism + grammaticality:gender:HL_home + grammaticality:gender:HL_social + (1| subject) + (1| item)*

**Table 2.3** Model output.

| **Model output for RTs in the SPRT - Region 6 adjective - Heritage** | | | | |
| --- | --- | --- | --- | --- |
| *Effect* | *df* | *Chisq* | *p* |  |
| grammaticality[grammatical] | 1 | .63 | .428 |  |
| gender[feminine] | 1 | .42 | .516 |  |
| dialang_z | 1 | 2.25 | .134 |  |
| bilingualism | 1 | 1.06 | .302 |  |
| HL_home_z | 1 | 1.07 | .300 |  |
| HL_social_z | 1 | .61 | .435 |  |
| grammaticality[grammatical]:gender[feminine] | 1 | .29 | .591 |  |
| grammaticality[grammatical]:dialang_z | 1 | 1.95 | .162 |  |
| grammaticality[grammatical]:bilingualism | 1 | 1.96 | .162 |  |
| grammaticality[grammatical]:HL_home_z | 1 | 2.00 | .157 |  |
| grammaticality[grammatical]:HL_social_z | 1 | 1.50 | .220 |  |
| gender[feminine]:dialang_z | 1 | 1.27 | .259 |  |
| gender[feminine]:bilingualism | 1 | .10 | .753 |  |
| gender[feminine]:HL_home_z | 1 | .49 | .486 |  |
| gender[feminine]:HL_social_z | 1 | .58 | .447 |  |
| grammaticality[grammatical]:gender[feminine]:dialang_z | 1 | 2.77 | .096 | + |
| grammaticality[grammatical]:gender[feminine]:bilingualism | 1 | 3.79 | .052 | + |
| grammaticality[grammatical]:gender[feminine]:HL_home_z | 1 | 1.12 | .290 |  |
| grammaticality[grammatical]:gender[feminine]:HL_social_z | 1 | .32 | .569 |  |
| Signif. codes: 0 ‘***’, 0.001 ‘**’, 0.01 ‘*’, 0.05 ‘+’, 0.1 ‘ ’ |  |  |  |  |

**Region 7: Post-critical region (spillover)**

**Between groups**

Model:

*(log)rt ~ grammaticality + group + gender + grammaticality:group + grammaticality:gender + group:gender + grammaticality:group:gender + (grammaticality| subject) + (grammaticality| item)*

**Table 2.4** Model output.

| **Model output for RTs in the SPRT - Region 7 spillover** | | | | |  |
| --- | --- | --- | --- | --- | --- |
| *Effect* | *df* | *Chisq* | *p* |  |  |
| grammaticality[grammatical] | 1 | 26.36 | **<.001** | ******* |  |
| group[heritage] | 1 | 21.98 | **<.001** | ******* |  |
| gender[feminine] | 1 | .55 | .457 |  |  |
| grammaticality[grammatical]:group[heritage] | 1 | 1.95 | **.162** |  |  |
| grammaticality[grammatical]:gender[feminine] | 1 | 3.78 | **.052** | **+** |  |
| group[heritage]:gender[feminine] | 1 | 3.10 | **.078** | **+** |  |
| grammaticality[grammatical]:group[heritage]:gender[feminine] | 1 | 5.94 | **.015** | ***** |  |
| Signif. codes: 0 ‘***’, 0.001 ‘**’, 0.01 ‘*’, 0.05 ‘+’, 0.1 ‘ ’ 1 |  |  |  |  |  |

**Heritage group**

Model:

*(log)rt ~ grammaticality + gender + proficiency + bilingualism + HL_home + HL_social + grammaticality:gender + grammaticality:proficiency + grammaticality:bilingualism + grammaticality:HL_home + grammaticality:HL_social + gender:proficiency + gender:bilingualism + gender:HL_home + gender:HL_social + grammaticality:gender:proficiency + grammaticality:gender:bilingualism + grammaticality:gender:HL_home + grammaticality:gender:HL_social + (1| subject) + (1| item)*

**Table 2.5** Model output.

| **Model output for RTs in the SPRT - Region 7 spillover - Heritage** | | | | |
| --- | --- | --- | --- | --- |
| *Effect* | *df* | *Chisq* | *p* |  |
| grammaticality[grammatical] | 1 | 6.20 | **.013** | ***** |
| gender[feminine] | 1 | .04 | .832 |  |
| dialang_z | 1 | .33 | .563 |  |
| bilingualism | 1 | .05 | .822 |  |
| HL_home_z | 1 | .15 | .703 |  |
| HL_social_z | 1 | 1.39 | .239 |  |
| grammaticality[grammatical]:gender[feminine] | 1 | 4.81 | **.028** | ***** |
| grammaticality[grammatical]:dialang_z | 1 | 1.62 | .204 |  |
| grammaticality[grammatical]:bilingualism | 1 | 2.89 | .089 |  |
| grammaticality[grammatical]:HL_home_z | 1 | 2.15 | .143 |  |
| grammaticality[grammatical]:HL_social_z | 1 | .99 | .320 |  |
| gender[feminine]:dialang_z | 1 | 5.21 | **.023** | ***** |
| gender[feminine]:bilingualism | 1 | 1.91 | .167 |  |
| gender[feminine]:HL_home_z | 1 | .37 | .542 |  |
| gender[feminine]:HL_social_z | 1 | .57 | .451 |  |
| grammaticality[grammatical]:gender[feminine]:dialang_z | 1 | .01 | .933 |  |
| grammaticality[grammatical]:gender[feminine]:bilingualism | 1 | .14 | .708 |  |
| grammaticality[grammatical]:gender[feminine]:HL_home_z | 1 | .19 | .661 |  |
| grammaticality[grammatical]:gender[feminine]:HL_social_z | 1 | .09 | .762 |  |
| Signif. codes: 0 ‘***’, 0.001 ‘**’, 0.01 ‘*’, 0.05 ‘+’, 0.1 ‘ ’ |  |  |  |  |

**3. Model specifications for the statistical analyses in section 4.2 (GJT)**

**Between groups**

Model:

*accuracy ~ group + grammaticality + gender + group:grammaticality + group:gender + grammaticality:gender + group:grammaticality:gender + (grammaticality + gender | subject) + (grammaticality | item)*

**Table 3.1** Model output.

| **Model output for accuracy in the GJT between groups** | | | | |  |
| --- | --- | --- | --- | --- | --- |
| *Effect* | *df* | *Chisq* | *p* |  |  |
| group[heritage speakers] | 1 | 31.91 | **<.001** | ******* |  |
| grammaticality[grammatical] | 1 | 62.99 | **<.001** | ******* |  |
| gender[feminine] | 1 | 21.45 | **<.001** | ******* |  |
| group[heritage speakers]:grammaticality[grammatical] | 1 | 24.12 | **<.001** | ******* |  |
| group[heritage speakers]:gender[feminine] | 1 | 1.61 | **.205** |  |  |
| grammaticality[grammatical]:gender[feminine] | 1 | .66 | **.416** |  |  |
| group[heritage speakers]:grammaticality[grammatical]:gender[feminine] | 1 | .97 | **.325** |  |  |

**Heritage group**

Model:

*accuracy ~ grammaticality + gender + dialang_z + bilingualism + HL_home_z + HL_social_z + grammaticality:gender + grammaticality:dialang_z + grammaticality:bilingualism + grammaticality:HL_home_z + grammaticality:HL_social_z + gender:dialang_z + gender:bilingualism + gender:HL_home_z + gender:HL_social_z + grammaticality:gender:dialang_z + grammaticality:gender:bilingualism + grammaticality:gender:HL_home_z + grammaticality:gender:HL_social_z + (1 | subject) + (1 | item)*

**Table 3.2** Model output.

| **Model output for accuracy in the GJT - Heritage** | | | | |
| --- | --- | --- | --- | --- |
| *Effect* | *df* | *Chisq* | *p* |  |
| grammaticality[grammatical] | 1 | 227.10 | **<.001** | ******* |
| gender[feminine] | 1 | 26.43 | **<.001** | ******* |
| dialang_z | 1 | 15.62 | **<.001** | ******* |
| bilingualism | 1 | 0.03 | .863 |  |
| HL_home_z | 1 | 0.25 | .618 |  |
| HL_social_z | 1 | 0.08 | .783 |  |
| grammaticality[grammatical]:gender[feminine] | 1 | 0.53 | .466 |  |
| grammaticality[grammatical]:dialang_z | 1 | 1.93 | .165 |  |
| grammaticality[grammatical]:bilingualism | 1 | 0.11 | .743 |  |
| grammaticality[grammatical]:HL_home_z | 1 | 28.20 | **<.001** | ******* |
| grammaticality[grammatical]:HL_social_z | 1 | 12.24 | **<.001** | ******* |
| gender:dialang_z | 1 | 1.28 | .257 |  |
| gender:bilingualism | 1 | 0.01 | .917 |  |
| gender:HL_home_z | 1 | 4.34 | **.037** | ***** |
| gender:HL_social_z | 1 | 2.78 | .095 | + |
| grammaticality[grammatical]:gender[feminine]:dialang_z | 1 | 4.51 | **.034** | ***** |
| grammaticality[grammatical]:gender[feminine]:bilingualism | 1 | 0.35 | .553 |  |
| grammaticality[grammatical]:gender[feminine]:HL_home_z | 1 | 0.53 | .466 |  |
| grammaticality[grammatical]:gender[feminine]:HL_social_z | 1 | 4.35 | **.037** | ***** |

**4.** **Gender Assignment Task (GAT)**

As illustrated in Figure 4 and Table 4.1, overall accuracy was very high in both heritage and homeland speakers showing that participants do not have difficulties with assigning the right gender to the nouns used in the tasks. Both groups were at ceiling in most of the conditions, only for *feminine* nouns, HSs’ mean accuracy score was 91%.


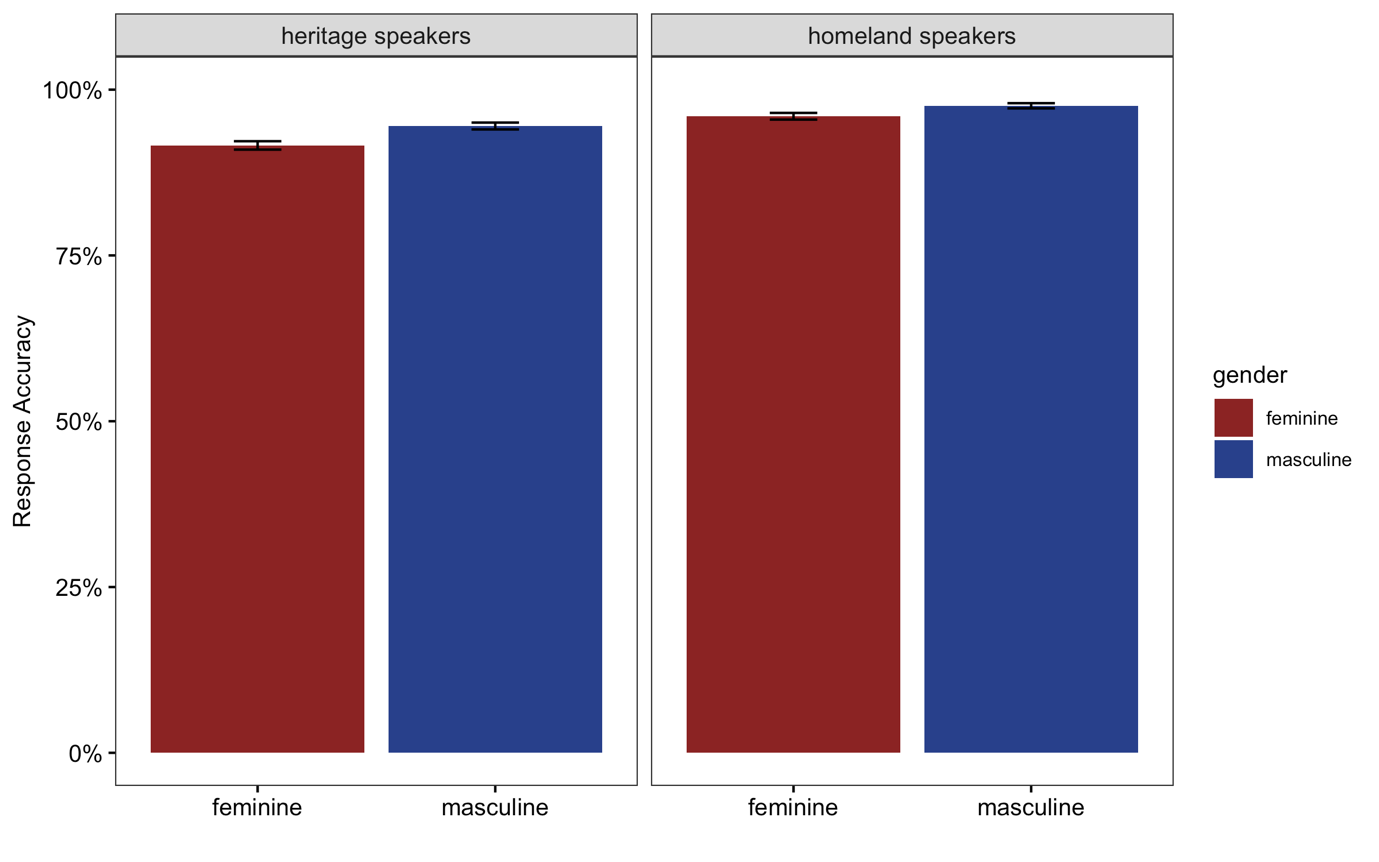


**Figure 4**. Overall accuracy in percentage for condition per group in the GAT. The bars represent the standard error to the mean.

**Table 4.1**. Mean accuracy scores (%) and standard deviations per condition for HSs and homeland speakers in the GAT.

|  | **Heritage speakers** | **Homeland speakers** |
| --- | --- | --- |
| **Noun** | M (*SD*) | M (*SD*) |
| *feminine* | 91 (*0.28*) | 96 (*0.19*) |
| *masculine* | 94 (*0.23*) | 97 (*0.15*) |

The model comparing groups found a main effect of *Group* (Chisq *=* 20.42, *p < .001*) indicating that HSs were less accurate compared to the homeland speakers. The main effect of *Gender* (Chisq *=* 5.31, *p = .021*) indicates that both groups were less accurate in assigning gender to feminine nouns as compared to masculine ones.

Model:

*accuracy ~ group + gender + group:gender + (gender | subject) + (1 | item)*

**Table 4.2** Model output.

| **Model output for accuracy in the GAT between groups** | | | | | |  |
| --- | --- | --- | --- | --- | --- | --- |
| *Effect* | *df* | *Chisq* | *p* |  |  |  |
| group [heritage speakers] | 1 | 20.42 | **<.001** | ******* |  |  |
| gender [feminine] | 1 | 5.31 | **.021** | ***** |  |  |
| group[heritage speakers]:gender[feminine] | 1 | .31 | 578 |  |  |  |

The model for the heritage group revealed a main effect of *Proficiency* (Chisq *=* 28.84, *p < .001*) indicating that the higher the score in the DIALANG, the better the accuracy in assigning gender. Furthermore, the significant interaction between *Gender:HL_home* (Chisq *=* 7.87, *p = .005*) indicates that the more the HL is used at home, the higher is the accuracy in assigning gender to masculine nouns, whereas this is not the case for feminine nouns.

Full model:

*accuracy ~ gender + dialang_z + bilingualism + HL_home_z + HL_social_z + gender:dialang_z + gender:bilingualism + gender:HL_home_z + gender:HL_social_z + (1 | subject) + (1 | item)*

**Table 4.3** Best fit model output.

| **Model output for accuracy in the GAT - Heritage** | | | | |
| --- | --- | --- | --- | --- |
| *Effect* | *df* | *Chisq* | *p* |  |
| gender[feminine] | 1 | 0.07 | .787 |  |
| dialang_z | 1 | 28.84 | **<.001** | ******* |
| bilingualism | 1 | 0.70 | .401 |  |
| HL_home_z | 1 | 0.60 | .440 |  |
| HL_social_z | 1 | 1.61 | .204 |  |
| gender[feminine]:dialang_z | 1 | 0.52 | .472 |  |
| gender[feminine]:bilingualism | 1 | 0.17 | .682 |  |
| gender[feminine]:HL_home_z | 1 | 7.87 | **.005** | ****** |
| gender[feminine]:HL_social_z | 1 | 0.99 | .319 |  |
